# Molecular Dynamics Simulations of a Cytochrome P450 from *Tepidiphilus thermophilus* (P450-TT) Reveal How Its Substrate-Binding Channel Opens

**DOI:** 10.3390/molecules26123614

**Published:** 2021-06-12

**Authors:** Abayomi S. Faponle, Anupom Roy, Ayodeji A. Adelegan, James W. Gauld

**Affiliations:** 1 Department of Biochemistry, Faculty of Basic Medical Sciences, Sagamu Campus, Olabisi Onabanjo University, Ago-Iwoye, Nigeria; asfb340@yahoo.com (A.S.F.); ayodejiadelegan@gmail.com (A.A.A.); 2 Department of Chemistry and Biochemistry, University of Windsor, Windsor, ON N9B3P4, Canada; roy11k@uwindsor.ca

**Keywords:** cytochrome P450, CYP, gating, P450-TT, CYP116B46, substrate channel, molecular dynamics simulations

## Abstract

Cytochrome P450s (P450) are important enzymes in biology with useful biochemical reactions in, for instance, drug and xenobiotics metabolisms, biotechnology, and health. Recently, the crystal structure of a new member of the CYP116B family has been resolved. This enzyme is a cytochrome P450 (CYP116B46) from *Tepidiphilus thermophilus* (P450-TT) and has potential for the oxy-functionalization of organic molecules such as fatty acids, terpenes, steroids, and statins. However, it was thought that the opening to its hitherto identified substrate channel was too small to allow organic molecules to enter. To investigate this, we performed molecular dynamics simulations on the enzyme. The results suggest that the crystal structure is not relaxed, possibly due to crystal packing effects, and that its tunnel structure is constrained. In addition, the simulations revealed two key amino acid residues at the mouth of the channel; a glutamyl and an arginyl. The glutamyl’s side chain tightens and relaxes the opening to the channel in conjunction with the arginyl’s, though the latter’s side chain is less dramatically changed after the initial relaxation of its conformations. Additionally, it was observed that the effect of increased temperature did not considerably affect the dynamics of the enzyme fold, including the relative solvent accessibility of the amino acid residues that make up the substrate channel wall even as compared to the changes that occurred at room temperature. Interestingly, the substrate channel became distinguishable as a prominent tunnel that is likely to accommodate small- to medium-sized organic molecules for bioconversions. That is, P450-TT has the ability to pass appropriate organic substrates to its active site through its elaborate substrate channel, and notably, is able to control or gate any molecules at the opening to this channel.

## 1. Introduction

Cytochrome P450 enzymes (P450s) are some of the most versatile and important known biocatalysts and have a diverse range of crucial functions [1,2,3,4,5,6], including cell signaling, detoxification, polyketide antibiotic production, bioremediation and more [7,8,9,10,11]. In addition, they often play central roles in the metabolism of exogenous compounds and pharmacological drugs in animals and humans. For example, CYP2D6 is a P450 isozyme expressed in the liver that has broad specificity and is responsible for the biotransformation of about 25% of all drugs used for therapeutic purposes [12].

Their diverse importance and utility are due to the fact that in general, P450s are capable of activating molecular oxygen, and thus, enable the incorporation of one of the oxygen atoms into the organic substrate via hydroxylation while the second oxygen atom is concomitantly reduced to water [3]. This occurs mostly, for instance, in the Phase I stage of drug metabolism prior to excretion [13]. However, P450s are also able to drive other reactions, including dealkylation, oxidation of heteroatoms, demethylation, epoxidation of a double bond, etc. [6].

Most P450s require two electrons and two protons to generate the active oxidant, which is a high-valent iron(IV)-oxo cationic radical intermediate (Cpd I), which transfers oxygen into the substrates during the catalytic cycle [1]. They are dependent on associated electron transfer proteins (e.g., cytochrome P450 reductase, ferredoxin reductase) that take reducing equivalents from reducing agents such as NAD(P)H and ferredoxin [14,15]. There exist certain P450s that possess closely linked or fused reducing partners (e.g., P450-Phthalate dioxygenase reductase [16,17,18] and P450-peroxygenases/peroxidases) that only require hydroperoxides, peracids, and other peroxy compounds such as oxygen atom donors to generate Cpd I via the “peroxide-shunt” mechanism [19,20,21]. Due to their self-sufficiency, they are assumed to be efficient biocatalysts for biotechnological/industrial applications as they do not require reducing partners for catalysis. Indeed, they bypass the two key electron transfers and protonation step in forming an iron(III)-hydroperoxo intermediate, which subsequently gets protonated to form Cpd I [22].

Recently, Class VII P450s have been identified that consist of highly efficient fused redox partners [18]. Furthermore, they have been shown to have interesting catalytic properties which include broad substrate specificity, chemical and extreme temperature tolerance (i.e., thermal stability), and good yields in a vector expression system. Notably, thermophilic P450 have been viewed as potential biocatalysts for industrial uses due to their chemical and thermal stability [23].

The CYP116B family of enzymes is a member of Class VII P450s. They possess a fused reductase domain containing an FMN and a 2Fe-2S prosthetic cluster [17,24], that is invariably turned-over when reducing equivalents are derived from NAD(P)H. The physiological roles of many CYP116B family members are still undetermined. However, it is now known that CYP116B1 can catalyze the oxidation of thiocarbamate herbicides [17], while CYP116B5 is involved in the oxidation of medium- to long-chain alkanes [18,25].

Recently, P450-TT (CYP116B46), a new member of the CYP116B family, from *Tepidiphilus thermophilus*, was found to hydroxylate, in an enantio- and regio-selective manner, the C5 of decanoic acid. This is an important step that precedes lactonization of the enantiomeric (*S*)-5-hydroxydecanoic acid formed to give (*S*)-δ-decalactone, a high-value fragrance chemical [26]. The capability of P450-TT to engage in oxy-functionalization was previously assumed when it was the first member of the CYP116B family to have its crystal structure resolved [27]. Remarkably, however, based on careful examination of the crystal structure, it was suggested that the opening to the substrate-binding channel is so narrow that even a molecule as small as methane would seem unable to gain access [27]. This stands in stark contrast to observations that P450-TT is capable of catalyzing reactions on the medium-alkyl chain decanoic acid [26]. Essential to understanding how P450-TT is able to perform its biological function(s), as well as to better understand its industrial application, is the knowledge of how it seemingly controls its channel access and, thus, its activity.

Herein, we describe a systematic molecular dynamics (MD) simulation-based investigation to examine the behavior of the substrate-binding channel and its residues; that is, to elucidate how P450-TT ensures molecular access to its active site via its substrate-binding channel.

## 2. Results

In the crystal structure of the native enzyme (PDB ID: 6GII), the opening of the putative substrate channel is quite narrow, as noted in the Introduction, at only ~1.7 Å, and stretches to ~14 Å into the enzyme [28]. This channel length is consistent with that observed in many other P450 enzymes. Notably, the opening to the substrate channel at the protein’s surface can be considered to consist of approximately nine amino acid residues. Of these, four can be classed as hydrophobic, three as polar (Gln66, Ser67, Asn87), and two as charged (Arg76, Arg88).

**Figure 1:**
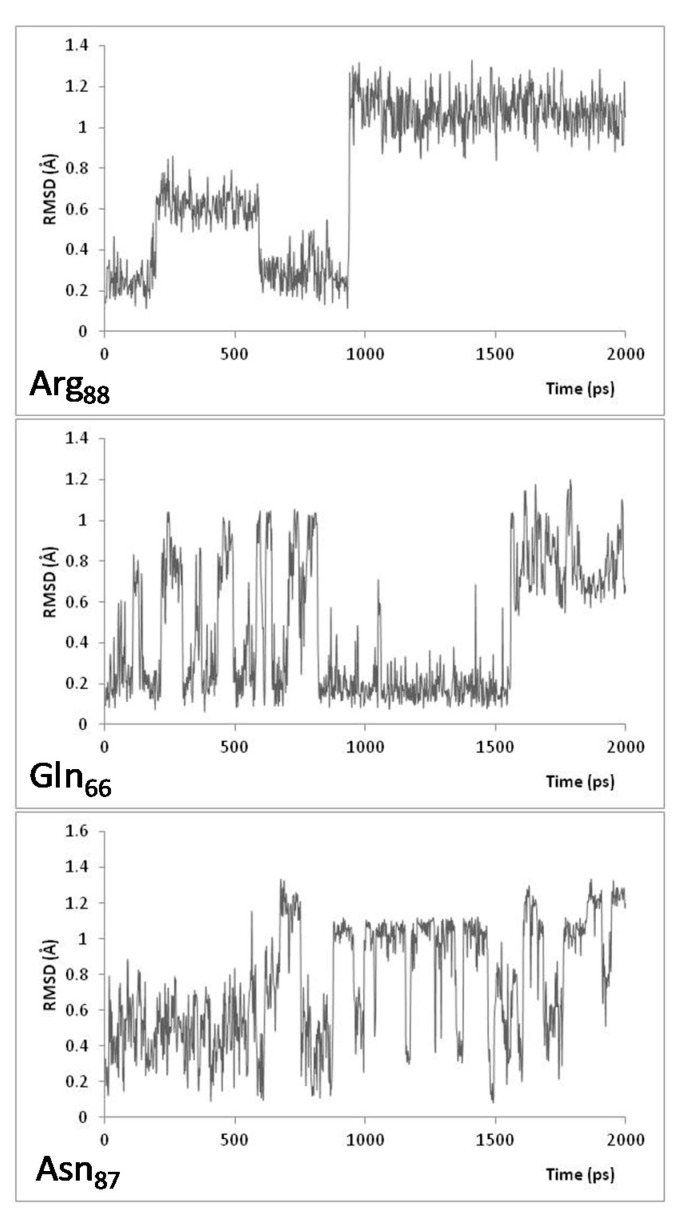
Plots of the Root Mean Square Deviation (RMSD) of Arg88, Gln66, and Asn87 over the course of 2 ns MD simulations, at 300 K, of the P450-TT enzyme.

**Figure 2:**
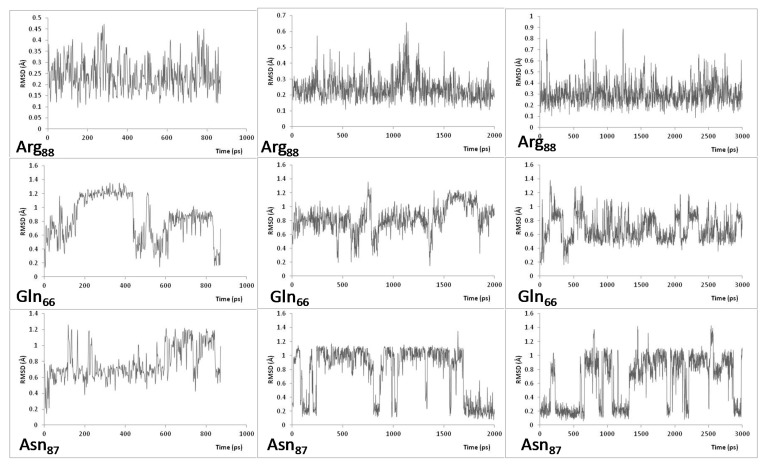
Plots of Root Mean Square Deviation (RMSD) of Arg88, Gln66, and Asn87 over the course of 1, 3, and 6 ns MD simulations, at 333 K, of the P450-TT enzyme.

**Figure 3:**
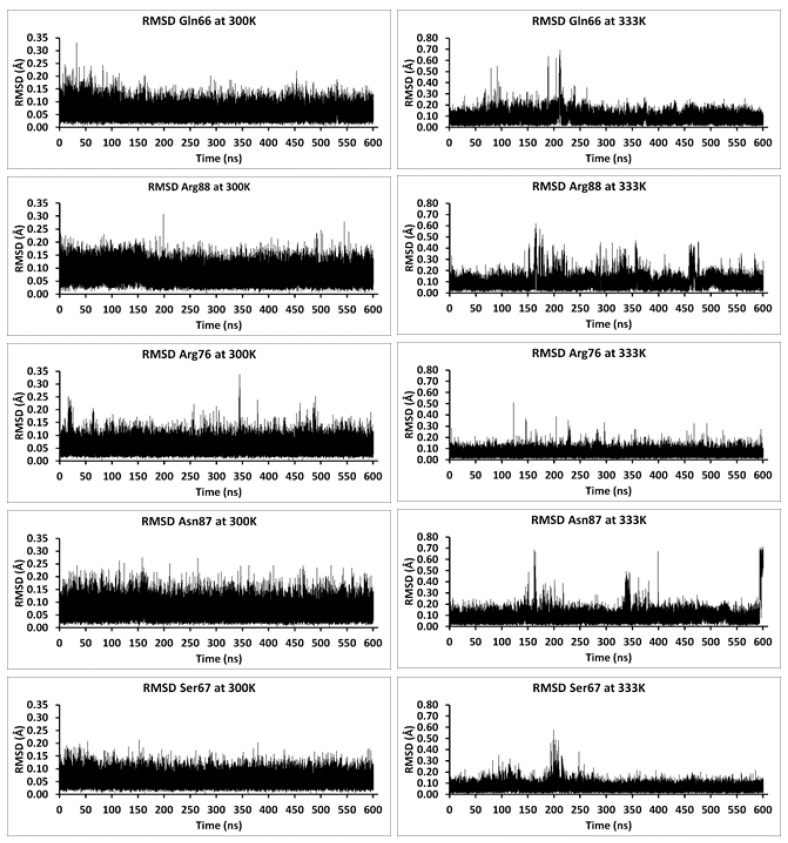
Plots of the Root Mean Square Deviation (RMSD) of Gln66, Arg88, Arg76, Asn87, and Ser67 residues over the course of the continuous 600.5 ns MD simulations of the P450-TT enzyme performed at 300 K and 333 K.

**Figure 4:**
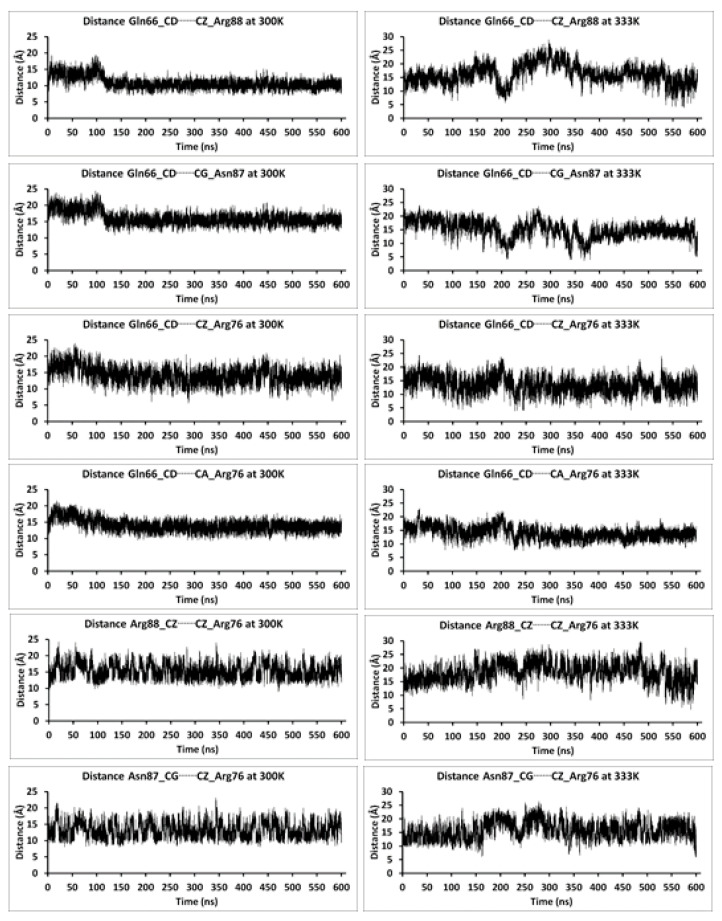
Selected measured distances (Angstrom) between Gln66-Arg88, Gln66-Asn87, Gln66-Arg76, Arg88-Arg76, and Asn87-Arg76 over the course of the continuous 600.5 ns MD simulations of P450-TT enzyme performed at 300 K and 333 K.

In order to examine conformational changes that may influence the width of the opening of putative substrate channel, multiple MD simulations (up to 600.5 ns and at 300 and 333 K) were performed, as described in the Methods section. The two temperatures were chosen due to the elevated temperatures of the natural environment of *T. thermophilus*. Since P450-TT is a thermophilic P450, it is possible that more significant conformational fluctuations of the substrate channel entrance amino acid residues may be observed. However, the most important conformational changes were generally also observed to occur during the first few nanoseconds of the MD simulations. The present discussion describes changes observed in both the short- (Figure 1 and Figure 2) and large-scale (Figure 3 and Figure 4) MD simulations.

Indeed, after initial minimization and MD simulations were performed on the protein structure, the channel opening was observed to increase to ~4.5 Å. Concomitantly, the measured area of the active site pocket of the minimized structure increased slightly from an initial value of 1492 Å^2^ to values in the range of 1531–1683 Å^2^ with a grid interval of 1.0 and at a cut-off distance of 11.5 Å to the surface of the substrate tunnel.

With respect to the residues involved in the channel and opening, it is noted that the hydrophobic residues of both the substrate channel and its entrance all exhibited negligible conformational variability (RMSD ranges) over the period of the MD simulations at both 300 and 333 K, even over the course of the 600.5 ns MD simulations; Figures S2 and S3. This also suggests that such residues have little dynamic-related effects on the other residues of the substrate channel entrance, at least at the two temperatures sampled. As a result, they were not considered further unless noted.

In contrast, however, considerably more significant conformational variability was observed for several of the charged and hydrophilic residues. For example, during the 600 ns MD simulation at 300 K (Figure 3), Gln66, Arg88, and Arg76 have RMSD values that range to almost 0.35 Å. The residues Asn87 and Ser67 also show large RMSD ranges at 300 K, though with markedly lower maximum RMSD values of <0.30 Å. Over the course of the 600.5 ns MD simulation at 333 K, significantly larger RMSD value ranges are observed for all five of the above hydrophilic residues with RMSD values as high as ~0.7 Å for Gln66 and Asn87. Hence, they may have a role(s) similar to that observed in some other P450s, wherein a number of amino acid residues at their substrate channel entrance participate in ligand recognition or in discrimination of potential ligands [28,29], which ultimately influence substrate specificity and catalytic mechanism.

Over the course of the 2 ns MD simulations performed at 300 K (Figure 1 and Figure 3), Gln66 and Arg88 exhibited conformational fluctuations characterized by a root mean squared deviation (RMSD) in the range ~0.1–1.2 Å (Figure 1). It is noted that during the course of the simulation, the positioning of Arg88 appears to change from a non-equilibration starting conformation to more stable state by the time it reaches ~1 ns (via a short-lived intermediate state from ~0.6–1 ns). This more stable state for Arg88 was confirmed by the 100 (Figure S12) and 600.5 ns (Figure 3) MD simulations. The possibility that Asn87 might also participate in the open–close mechanism of the channel’s entrance was considered. However, its side chain was observed to be positioned between the hydrophilic side chains of Tyr236 and Ser235. Thus, its observed RMSD behavior may be in part due to a less definitively preferred binding position.

In the MD simulations performed at 333 K (Figure 2 and Figure 3), the most significant residue conformational fluctuations were observed for the hydrophilic residues Gln66 and Arg88 (Figure 2 and Figure 3). This likely reflects, at least in part, that their side chains are not impeded by interactions (steric or chemical) with other residues, and thus, can move more freely. In contrast, the side chain of Asn87 was again observed to be sequestered between the hydrophilic side chains of Tyr236 and Ser235. Thus, its movement is limited, as evidenced by its considerably reduced conformational variability, and would only move within this entrapment, which gave reversed trajectories (down spiking seen in both Figure 1 and Figure 2). However, in the 600.5 ns MD simulations at both 300 and 333 K, the side chain of Asn87 was observed to rotate toward Arg76 from 594 ns to 600 ns (Figure 3).

Gln66 and Arg88 seem to play important roles in the tightening (closing) and relaxation (opening) of the entrance to the substrate channel. In the minimized conformation, the groove between the two residues was measured to have a gap of 83.8 Å^3^ in volumetric terms (with a measured area of 99.3 Å^2^).

This suggests that the gap may only narrow once the enzyme is relaxed back to its native conformation and as long as it is not in the native state, the entrance remains open and can accommodate appropriate organic molecules to be chemically converted to products. The dynamic actions of the amino acids Gln66 and Arg88 in the enzyme serve as the gating mechanism that controls access of ligands to the active site through the substrate tunnel [30,31].

**Figure 5:**
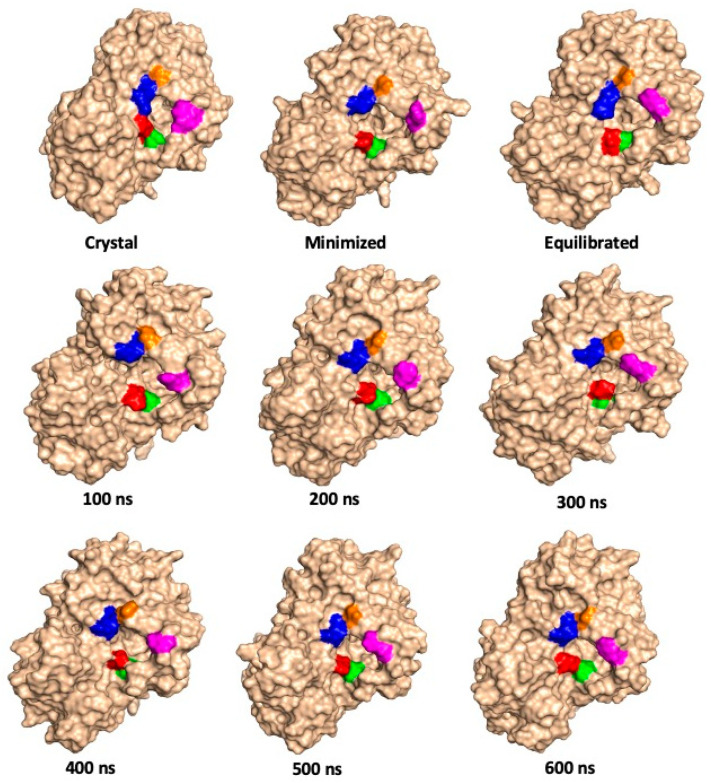
Illustration of the position of selected hydrophilic residues of P450-TT enzyme involved in the substrate channel, as observed in the X-ray crystal structure, minimized and equilibrated structures (see Methods), and at periodic times during the 600.5 ns MD simulations, performed at 300 K. Color code: Gln66 = red; Ser67 = green; Arg76 = magenta; Arg88 = blue, and Asn87 = orange.

**Figure 6:**
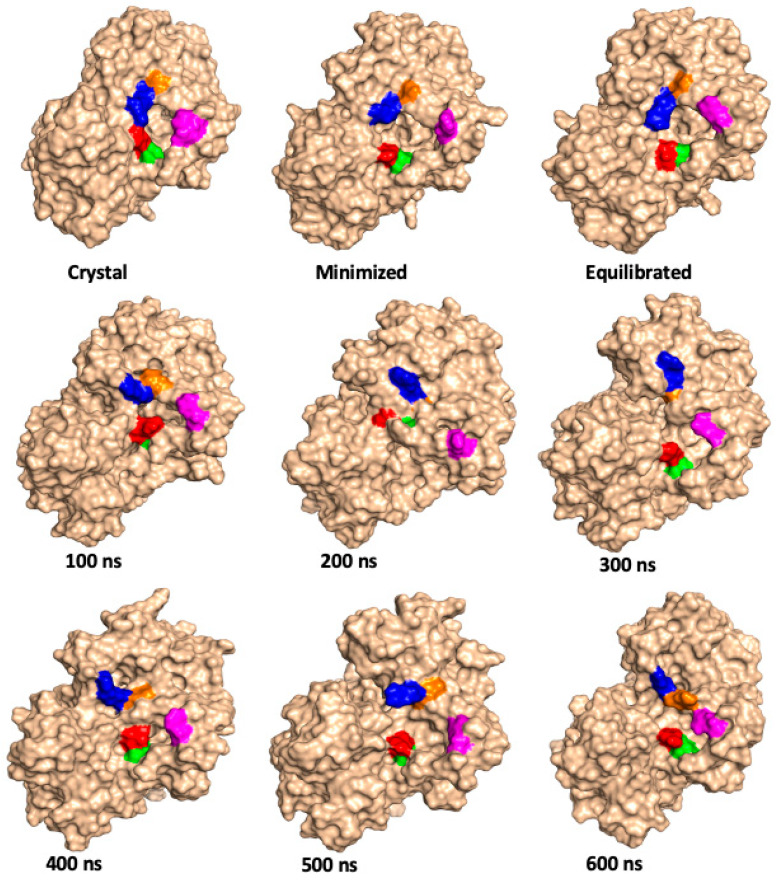
Illustration of the position of selected hydrophilic residues of the P450-TT enzyme involved in the substrate channel, as observed in the X-ray crystal structure, minimized and equilibrated structures (see Methods), and at periodic times during the 600.5 ns MD simulations, performed at 333 K. Color code: Gln66 = red; Ser67 = green; Arg76 = magenta; Arg88 = blue, and Asn87 = orange.

In X-ray crystallographic structures, protein residues may be, for example, more densely packed than they are in a solution [32]. In the X-ray crystal structure of P450-TT (see Methods), the distance between Gln66 and Arg88 is ~5.91 Å and these residues’ surface figures (Figure 5 and Figure 6) shows that the substrate channel is tightly closed. This was also noted in the Introduction. However, upon minimization and equilibration, the distance between these residues increases to 11.21 Å and 11.43 Å at 300 K and 333 K, respectively. This suggests that the substrate channel is more open (see below).

In the short (progressively discontinuous) MD simulations, the distances were measured between key interacting channel residues that form two protrusions (otherwise referred to as an upper and a lower contour) within the substrate channel itself. For example, the residues Trp198, Gln242, and Phe86 create the upper contour. Notably, in MD simulations at both 300 and 333 K, these protrusions were observed to shift positions slightly, resulting in a narrowing of the substrate channel (Figures S5, S7–S9). For example, the distances between HD11 of Leu65 and HD2 of Phe86 (i.e., the nearest distance between hydrogens on the side chain methyl of Leu65 and phenyl hydrogen of Phe86) at 300 and 333 K were both within 0.4 Å of each other and approximately 8.7 Å, although the cut-off distances of the isoelectric surfaces were 11.5 Å and 10.5 Å, respectively. Significantly, in all MD simulations at both 300 and 333 K, a very similar narrowing of the substrate channel was observed that would still allow potential putative substrate molecules to pass through.

**Figure 7:**
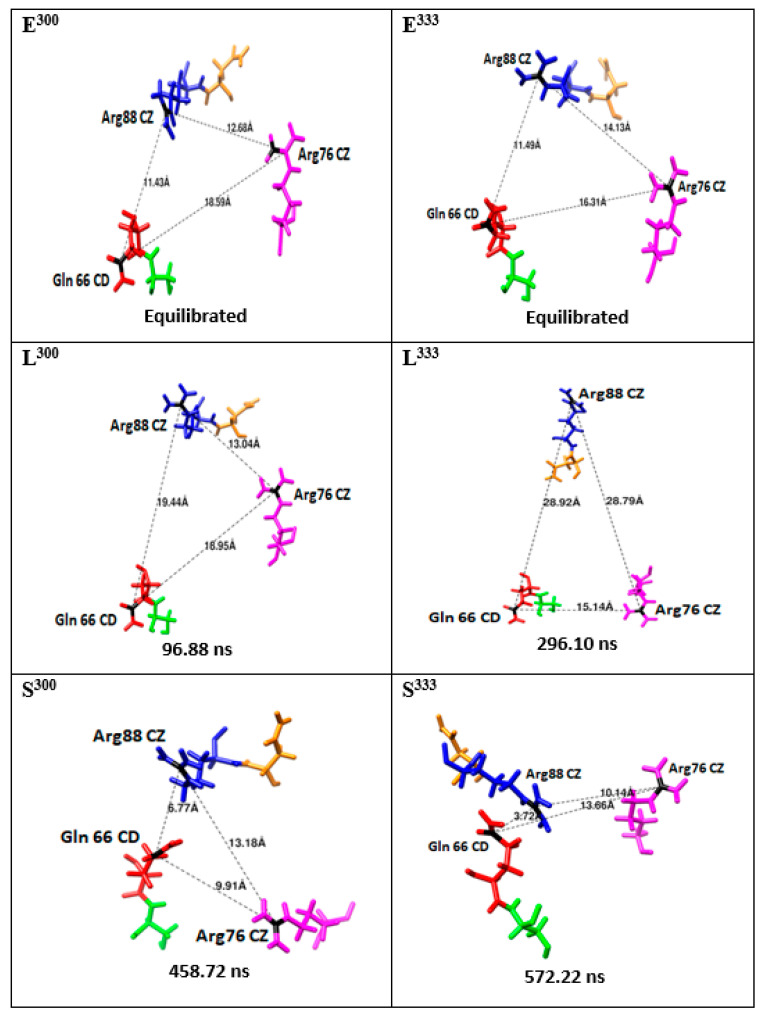
Inter-residue distances between key substrate channel entrance residues of P450-TT enzyme, over the course of the 600.5 ns MD simulations at 300 and 333 K, showing their distances at equilibration (E^300^ and E^333^), and their longest (L^300^ and L^333^) and shortest (S^300^ and S^333^) distances.

The distances between key substrate channel residues were also observed over the course of the 600.5 ns MD simulations performed at 300 and 333 K (Figure 4 and Figure S3). Some general observations were made over the course of these MD simulations. In particular, at 300 K, the distance between Gln66 and Arg88 is observed to fluctuate; significantly increasing during the first 100 ns, then decreasing. Meanwhile, at 333 K, the distance between Gln66 and Arg88 is observed to fluctuate considerably more (Figure 4); the distance is greatly increased at ~160 ns, decreased at ~210 ns, increased at ~300 ns, and then significantly decreased at ~570 ns. This suggests that there are some dynamics to the opening of the substrate channel; more open and perhaps less open substrate channels. This ‘opening and closing’ of the substrate channel is in part also illustrated by the distances observed between these residues. At equilibration at 300 and 333 K, the inter-residues distances between Gln66 and Arg88 are 11.43 Å and 11.49 Å, respectively (Figure 7). In contrast, the largest distances observed between these residues during the course of the 600.5 ns MD simulations are 19.44 Å (300 K) and 28.92 Å (333 K) and occur at 96.88 ns and 296.10 ns, respectively. However, the shortest distances observed between these residues during the course of the 600.5 ns MD simulations are just 6.77 Å (300 K) and 3.72 Å (333 K) and occur at 458.72 ns and 572.22 ns, respectively. It is noted that these shortest observed distances are within 1–2 Å of their inter-residue distance within the X-ray crystal structure (see Methods). It is noted that in the 600.5 ns MD simulation at 333 K, analogous increasing and decreasing inter-residue distances are observed for Arg88 and Asn87 (Figure 4). For example, the Gln66…Asn87 distance is also observed to, at times, have significantly decreased to 4.03 Å (~211 ns), 3.90 Å (~342 ns), 3.87 Å (~381 ns), and 4.93 Å (~600 ns). This suggests that Asn87 may also be able to play a role in gating of the substrate channel.

The surfaces, and dynamics, of the substrate channel residues Gln66, Ser67, Arg76, Asn87, and Arg88 over the course of the 600.5 ns MD simulations at 300 and 333 K are shown in Figure 5 and Figure 6, respectively. Again, one can see the fluctuating distance between, for example, Gln66 (red) and Arg88 (blue) in the X-ray crystal structure and over the course of the MD simulations and further suggests a quite dynamic substrate channel opening.

We examined the solvent exposure of selected residues that contribute to the substrate tunnel in various structures used (e.g., the X-ray crystal structure) and obtained (e.g., over the course of the 600.5 ns MD simulations at 300 and 333 K). More specifically, we calculated their relative solvent accessibility (RSA). RSA, a measure of the extent of exposure of residue to solvent, is given by the ratio of solvent accessible surface area to the maximum possible solvent accessible surface area (maxASA) for the particular residue. While maxASA values are at times taken from those calculated using the tripeptide Gly-X-Gly, where X is the residue in question, in the present study, we have chosen to use as reference values those derived by Tien et al. [33]. For simplicity, herein only the RSA values calculated for selected substrate channel residues in the X-ray crystal structure, minimized and equilibrated structures, and snapshots taken every 100 ns (i.e., at 100 ns, 200 ns, 300 ns, 400 ns, 500 ns, and 600 ns) of the 600.5 ns MD simulations performed at 300 K and 333 K are shown in Table 1. These structures were chosen to highlight the changes observed going from the native state (equilibrated) structure to the ones in which the channel is open and to also consider the effects of an increased temperature.

In the equilibrated structure, or that in which the channel is narrow, the low RSA values of the residues within the channel suggest that the tightness or narrowness of the tunnel plays a role in minimizing their exposure to solvent molecules. In the equilibrated structure at 300 K, Arg88 is predicted to have the largest observed RSA value (0.55), while at 333 K, Gln66 was predicted to have the largest observed RSA value (0.42). These values also likely reflect their locations at the entrance, where they are considerably exposed to the external environment. However, over the course of the MD simulation at 300 K, while the RSA value of Gln66 ranges from 0.35–0.50, that of Arg88 barely varies, ranging from 0.36–0.38. In contrast, at 333 K, Gln66 still shows the greatest variation but it now ranges from 0.13–0.60. Moreover, Arg88 now exhibits a larger range from 0.32–0.80. It is also noted that the calculated RSA values of both Gln66 and Arg88 increase and decrease over the course of the simulations; that is, they do not simply monotonically increase or decrease. This further supports a dynamic substrate channel in P450-TT. It is noted that at 300 K, the calculated RSA values of the hydrophobic residues (Leu65, Phe68, Phe86, Val91, Val246, and Phe397) in general decrease markedly after the first 100 ns. In contrast, at 333 K, several of these residues (i.e., Leu65, Phe86, and Val91) are predicted to have sizeable RSA values over the course of the simulation.

These changes are indicative of the dynamic roles these residues play in relaxing the channel opening obviously with the side chain of Gln66, supposedly acting like a “swinging door” [30]. In general, increasing and decreasing RSA values are observed for all putative residues of the substrate tunnel. This further suggests that it may be possible for P450-TT to allow or accommodate a broader range of substrates than has been previously suggested [27]. In fact, we docked the putative substrate decanoic acid to the P450-TT protein conformation obtained at 100 ns (i.e., when the substrate channel was at one of its observed most open conformations) during the 300 K MD simulation in order to see if the open substrate channel may be able to accommodate such a sized substrate (Figures S17 and S18). Importantly, two docked complexes were obtained with binding affinities of −3.5 and −3.3 kcal mol^−1^, in which the putative substrate was well accommodated within the substrate channel in the vicinity of its entrance. Indeed, it has been experimentally shown that P450-TT can catalyze the conversion of decanoic acid to (S)-δ-decalactone within its substrate cavity [26].

## 3. Methods

All calculations were performed using the Gaussian 09 [34] and AMBER16 [35] programs. A suitable X-ray crystal structure of P450-TT, with 1.9 Å resolution, was obtained from the protein data bank (PDB ID: 6GII) [27]. Subsequently, the protein was protonated with the online webserver H++ [36,37,38] at pH 7. Of the titratable amino acid residues, arginine and lysine were chosen to be protonated while all negatively charged glutamate and aspartate were left deprotonated. Of the histidyls, six (His98, 113, 266, 327, 331, 402) were protonated with the others left neutral (i.e., His356(δ-NH) or His2, 117, 155, 174, 238, 249, 348, 381, 393(ε-NH)). It should be noted that the residues have been renumbered during the initial setup with Ambertools, see the Supporting Information. As such, the total charge of the system which also includes the manually created heme-iron-oxo complex is −5.

To create the parameters for the heme, geometry optimizations and electrostatic potential charge fitting were done using Gaussian 09 at the UB3LYP [39,40]/BS level of theory, where BS is the 6-31G(d) [41,42] basis set on all atoms except iron, which has the double-ζ quality LANL2DZ ECP basis set [43]. The heme center resembles the typical high-valent iron(IV)-oxo cationic radical intermediate, Cpd I, with an axial cysteinyl ligand [1]. The parameters and topologies for the P450-TT setup were derived following the protocol outlined for metalloenzymes using the metal center parameter builder (mcpb.py version) [44], as implemented in Ambertools.

The resulting system was solvated using a TIP3P water model within a truncated octahedron waterbox radius of 12 Å and neutralized with five sodium ions. All crystal waters were retained while the one bound to the heme iron was fully deprotonated to form the iron-bound oxo atom of Cpd I. The solvated P450-TT enzyme setup contains a total of 54,283 atoms, which includes 15,830 molecules of TIP3P water. Solvation was followed by a series of minimization steps, which included minimizations of the hydrogen atoms on all heavy atoms using the SHAKE algorithm, the protein backbone, and all heavy atoms except the heme-iron Cpd I cofactor. The TIP3P waters were energy-minimized then heated to 300 K using Langevin dynamics with the collision frequency, *γ*_ln = 2 and at a constant pressure with periodic boundary conditions, ntp = 1. For the progressively discontinuous (see below) molecular dynamic (MD) simulations (i.e., 1, 2, 3, 6 and 100 ns), an equilibration period of 20 ps was used. For the markedly longer 600 ns MD simulations, an equilibration period of 500 ps was used. Finally, all atoms, including water molecules, were minimized while Cpd I was held constrained. Molecular dynamics (MD) simulations were then performed on the minimized system on the scale of nanoseconds (1, 2, 3, 6, and 100 ns) at 300 K, and then again at 333 K in order to observe the effects of increased temperature. As noted above, these MD simulations were progressively discontinuous, with each (except the first one for which the minimized structure was used) being performed from the last structure of the previous simulation. That is, the reference state of the subsequent MD simulation was the last conformation of the previous. However, for the 600.5 ns MD simulations, the minimized structure was used. Thus, in effect, multiple, non-dependent MD simulations were run on the systems.

All Docking calculations were performed using Autodock Vina [45]. The P450-TT structure(s) used, with relaxed (i.e., open) substrate channel(s), were chosen from the 600.5 ns MD simulations; specifically, the protein conformation obtained at 100 ns was selected. The ligand decanoic acid (capric acid) was used as a substrate model and optimized prior to being docked. In addition, the TIP3P water and Na^+^ counter ions were removed from the selected solvated protein conformation(s) and Kollman charges addition reestablished the total system charge of −5. A grid box configuration of 26 × 36 × 30 with 0.814 Å spacing was prepared and was adjusted to cover the entrance of the substrate channel and extended to the catalytically important heme region. That is, only possible bindings of the substrate in and around the substrate channel and its entrance was considered.

## 4. Conclusions

In this present study, we have performed a series of molecular dynamics (MD) simulations at 300 and 330 K to examine the dynamic behavior of residues involved in the substrate channel to the active site of a new cytochrome P450 member of the CYP116B family from *Tepidiphilus thermophilus*; P450-TT. In addition, we have also docked a putative substrate, in part to help determine if it could indeed fit within the substrate channel, and have calculated the Relative Solvent Accessibility (RSA) values for a number of residues that are thought to be involved in the substrate channel itself and/or opening.

Importantly, the present results suggest that the substrate channel entrance observed in the native X-ray crystallographic structure (PDB ID: 6GII) is not in fact the maximum channel opening possible. Rather, the residues of the substrate channel entrance show a considerable dynamic behavior. For example, in the above X-ray crystal structure, the inter-residue distance between the putative channel entrance residues Gln66 and Arg88 is ~5.91 Å. At 300 K; this distance is observed to possibly vary over the range of 6.77 Å to 19.44 Å, while at 333 K it ranges from 3.72 Å to 28.92 Å. That is, for the first time, the present results reveal the key dynamic behavior of the residues Gln66 and Arg88 in controlling the opening to the substrate channel. The present simulations suggest a possible “swinging door”-type mechanism in which both Arg88 and Gln66 change conformation.

As noted, RAS values were calculated for a number of hydrophilic and hydrophobic residues thought to be involved in the substrate channel and/or entrance. The observed variabilities in several of their values further support a possible dynamicism to the positioning of the residues over the course of the simulations at both 300 and 333 K, and a possible temperature dependence.

## Figures and Tables

**Table 1:** Relative solvent accessibility of some residues of the substrate channel.

Relative Solvent Accessibility (RAS) at 300 K									
Residues	Crystal Structure	Minimized Structure	Equilibrated Structure	100 ns	200 ns	300 ns	400 ns	500 ns	600 ns
Leu65	0.31	0.32	0.36	0.38	0.23	0.25	0.26	0.23	0.24
Gln66	0.27	0.40	0.26	0.50	0.46	0.48	0.35	0.41	0.38
Ser67	0.26	0.20	0.21	0.20	0.14	0.15	0.16	0.22	0.27
Phe68	0.15	0.16	0.20	0.20	0.09	0.08	0.11	0.13	0.14
Arg76	0.58	0.55	0.40	0.62	0.51	0.56	0.40	0.49	0.49
Asn87	0.22	0.27	0.26	0.47	0.35	0.42	0.57	0.34	0.35
Arg88	0.47	0.39	0.55	0.38	0.36	0.36	0.36	0.37	0.36
Val91	0.27	0.12	0.14	0.35	0.11	0.16	0.17	0.17	0.14
Val246	0.25	0.26	0.21	0.11	0.03	0.02	0.03	0.03	0.01
Phe397	0.05	0.03	0.04	0.06	0.07	0.04	0.09	0.06	0.08
**Relative Solvent Accessibility (RAS) at 333 K**									
**Residues**	**Crystal Structure**	**Minimized** **Structure**	**Equilibrated** **Structure**	**100 ns**	**200 ns**	**300 ns**	**400 ns**	**500 ns**	**600 ns**
Leu65	0.31	0.32	0.32	0.39	0.11	0.27	0.20	0.24	0.22
Gln66	0.27	0.40	0.42	0.60	0.13	0.42	0.26	0.38	0.54
Ser67	0.26	0.20	0.23	0.30	0.30	0.16	0.20	0.13	0.16
Phe68	0.15	0.16	0.23	0.42	0.56	0.17	0.14	0.11	0.22
Arg76	0.58	0.55	0.48	0.38	0.38	0.59	0.57	0.31	0.46
Phe86	0.32	0.45	0.32	0.34	0.32	0.59	0.38	0.31	0.17
Asn87	0.22	0.27	0.23	0.56	0.05	0.15	0.18	0.20	0.65
Arg88	0.47	0.39	0.35	0.40	0.41	0.32	0.52	0.80	0.46
Val91	0.27	0.12	0.18	0.34	0.36	0.78	0.14	0.28	0.26
Val246	0.25	0.26	0.14	0.32	0.11	0.27	0.04	0.02	0.04
Phe397	0.05	0.03	0.03	0.14	0.02	0.04	0.05	0.05	0.06

## Data Availability

Data can be made available upon written request to the corresponding author.

## Supplementary Materials

The following are available online, Figures S1–S18, Additional plots of RMSD, at 300 and 333 K, of various amino acid residues positioned at or near the substrate channel opening or in the substrate channel.
