# Supplementary material for: Molecular Dynamics Simulations of a Cytochrome P450 from Tepidiphilus thermophilus (P450-TT) Reveal How Its Substrate-Binding Channel Opens
Source: Molecules. 2021 Jun 12;26(12):3614. doi: 10.3390/molecules26123614 (PMC8231624; doi:10.3390/molecules26123614)
Supplement: Supplementary file 1 [file molecules-26-03614-s001.zip › molecules-1192556-supplementary.pdf]

Article

Molecular Dynamics Simulations of a Cytochrome P450 from *Tepidiphilus thermophilus* (P450-TT) Reveal How Its Substrate-Binding Channel Opens

Abayomi S. Faponle[1], Anupom Roy[2], Ayodeji A. Adelegan[1] and James W. Gauld[2*]

[1] Department of Biochemistry, Faculty of Basic Medical Sciences, Sagamu Campus, Olabisi Onabanjo University, Ago-Iwoye, Nigeria.

[2] Department of Chemistry and Biochemistry, University of Windsor, Windsor, ON N9B3P4, Canada; asfb340@yahoo.com (A.S.F); roy11k@uwindsor.ca (A.R.); ayodejiadelegan@gmail.com (A.A.A)

* Correspondence: gauld@uwindsor.ca; Tel.: +1-519-253-3000 (ext. 3992); Fax: +1-519-973-7098

Supporting Information

Total pages: 19

(Figures S1-S18)

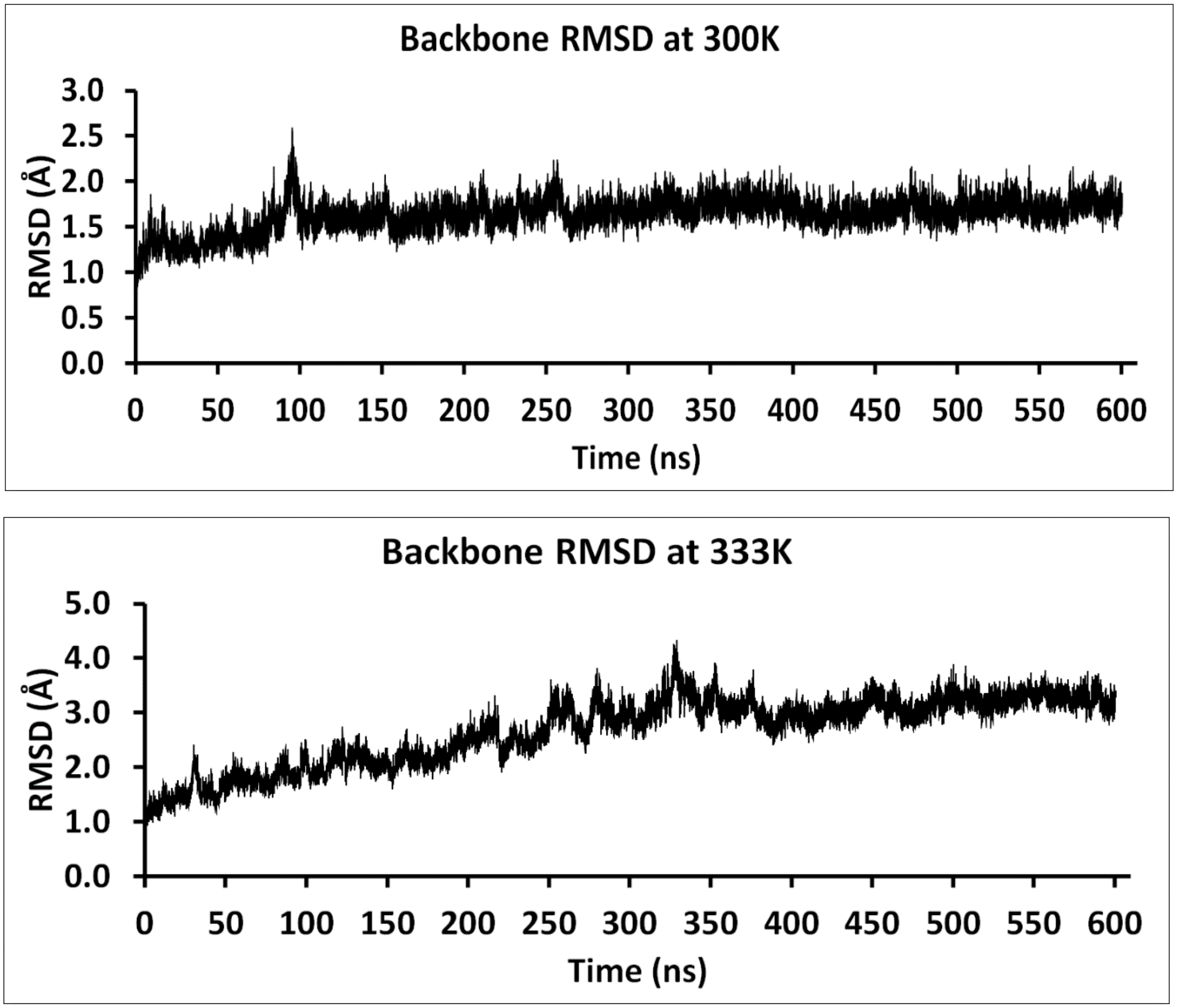

FigureS1: Backbone RMSD at 600 ns MD simulations of p450-TT enzyme as observed at 300K and 333K.

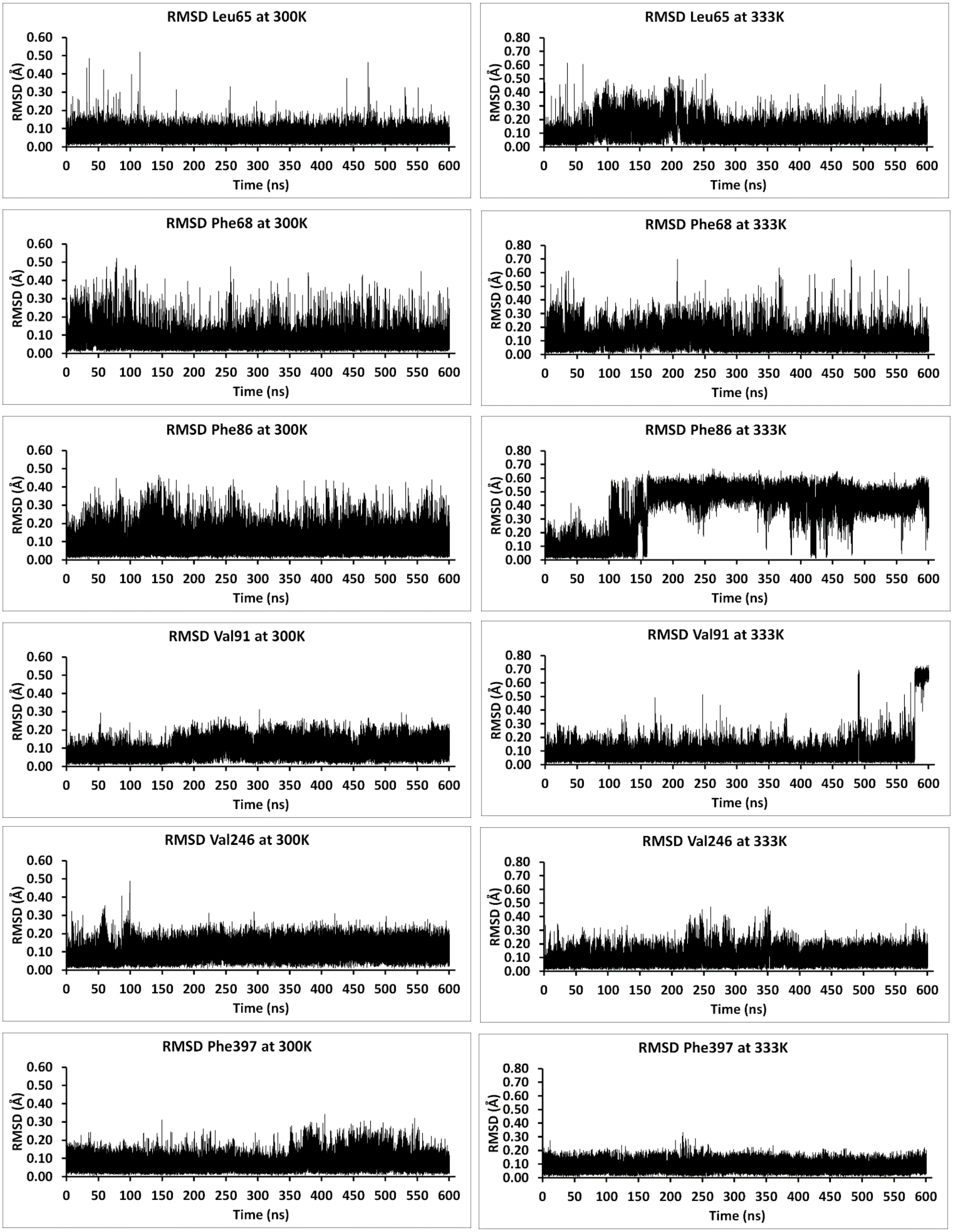

FigureS2: RMSD of Leu65, Phe68, Phe86, Val91, Val246, and Phe397 residues at 600.5 ns MD simulations of p450-TT enzyme as observed at 300K and 333K

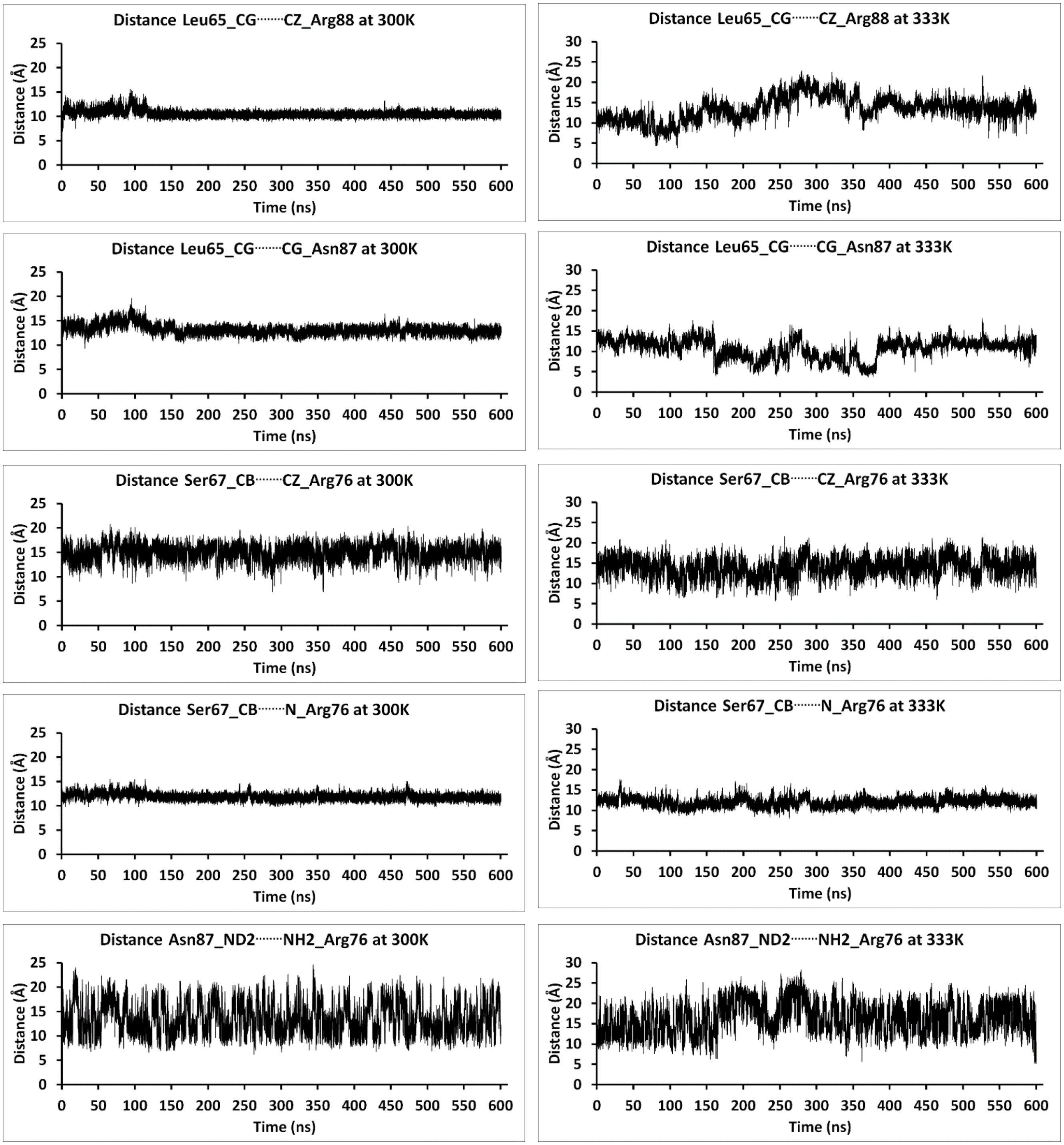

FigureS3: Measured distances of Leu65-Arg88, Leu65-Asn87, Ser67-Arg76, and Asn87-Arg76 at 600.5 ns MD simulations of p450-TT enzyme as observed at 300K and 333K.

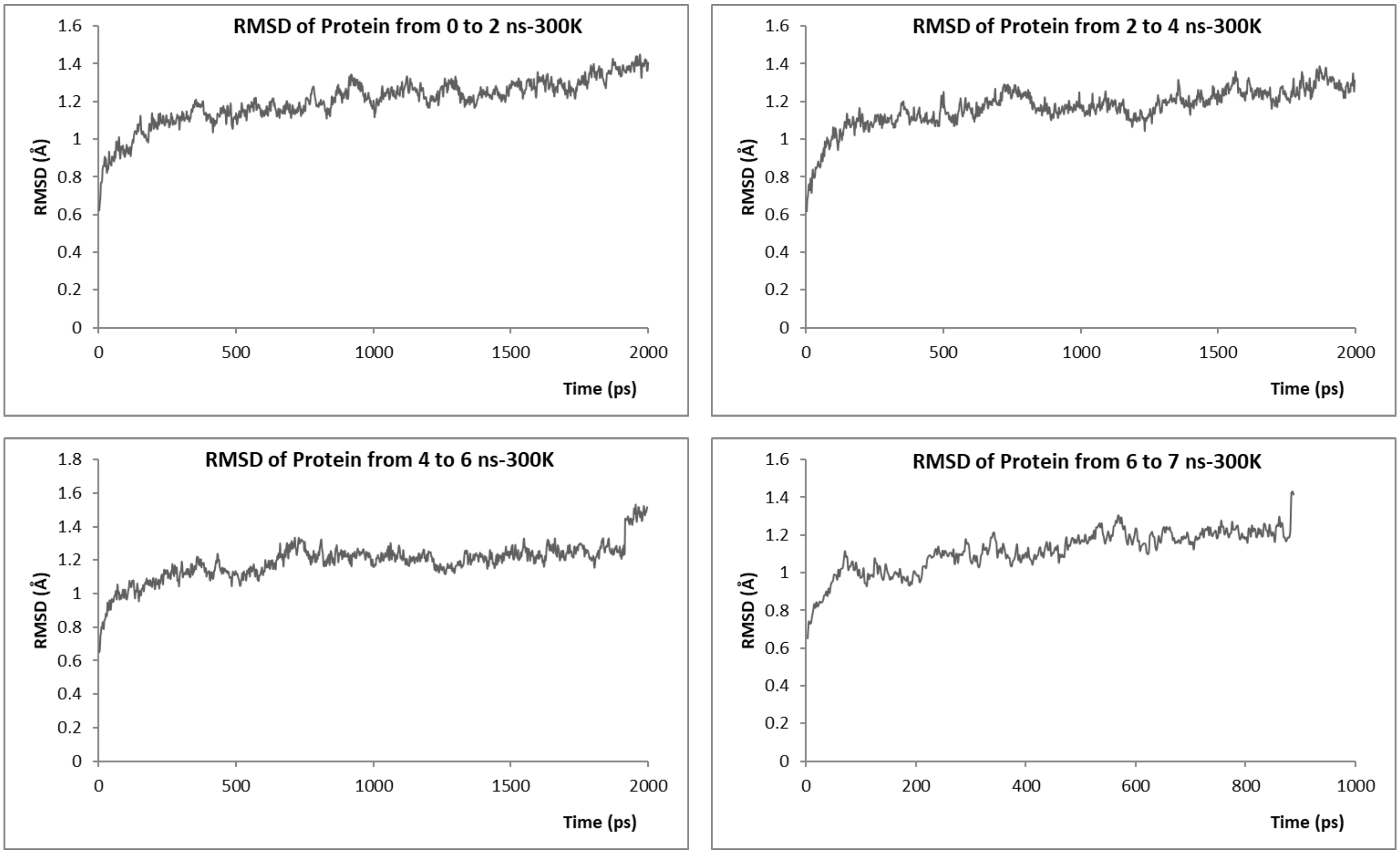

Figure S4: Root mean square deviation of protein from 0 to 2, 2 to 4, 4 to 6, and 6 to 7 ns MD simulations of P450-TT enzyme as observed at 300 K.

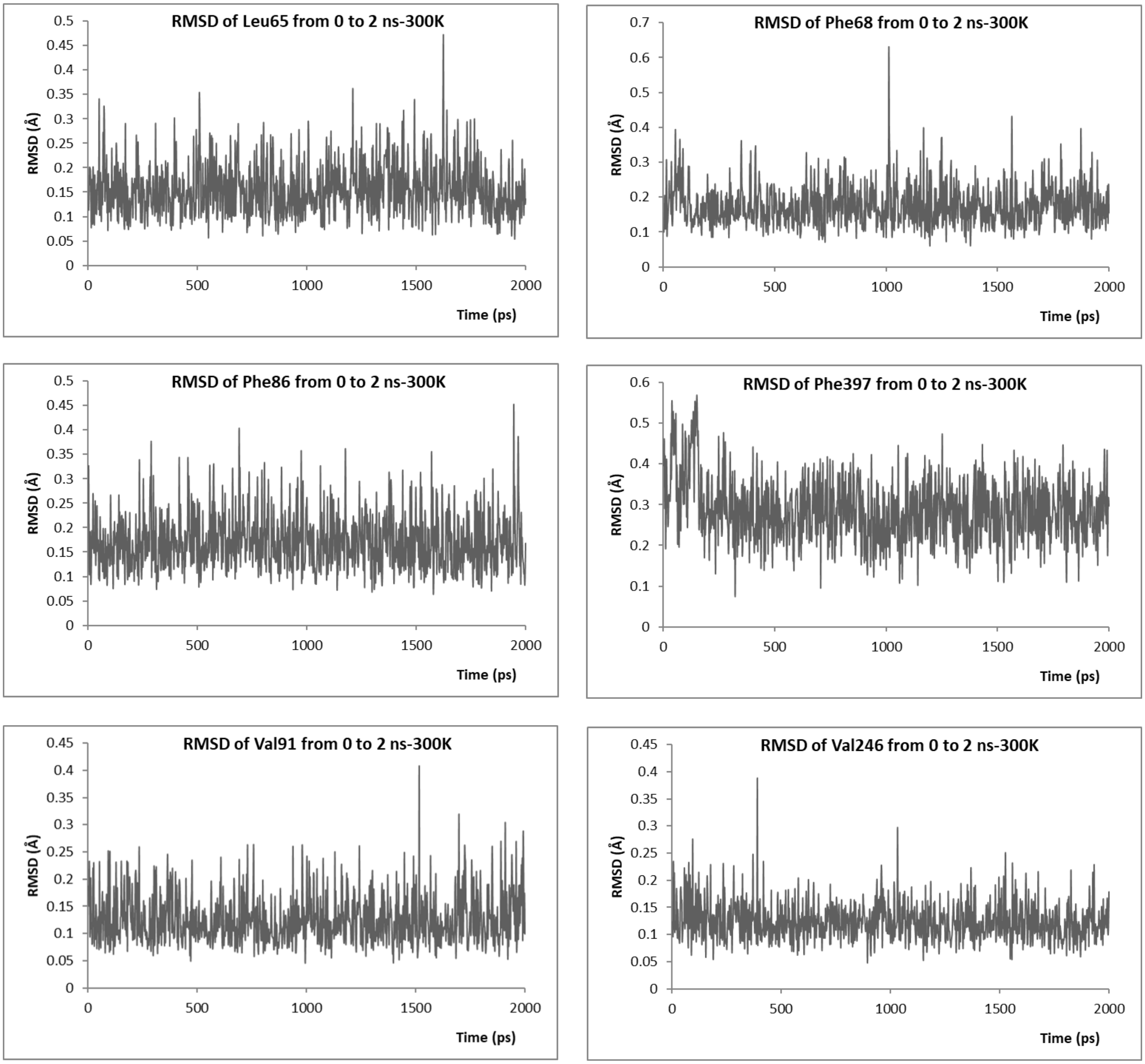

Figure S5: Root mean square deviation of Leu65, Phe68, Phe86, Phe397, Val91 and Val246 from 0 to 2 ns MD simulations of P450-TT enzyme as observed at 300 K.

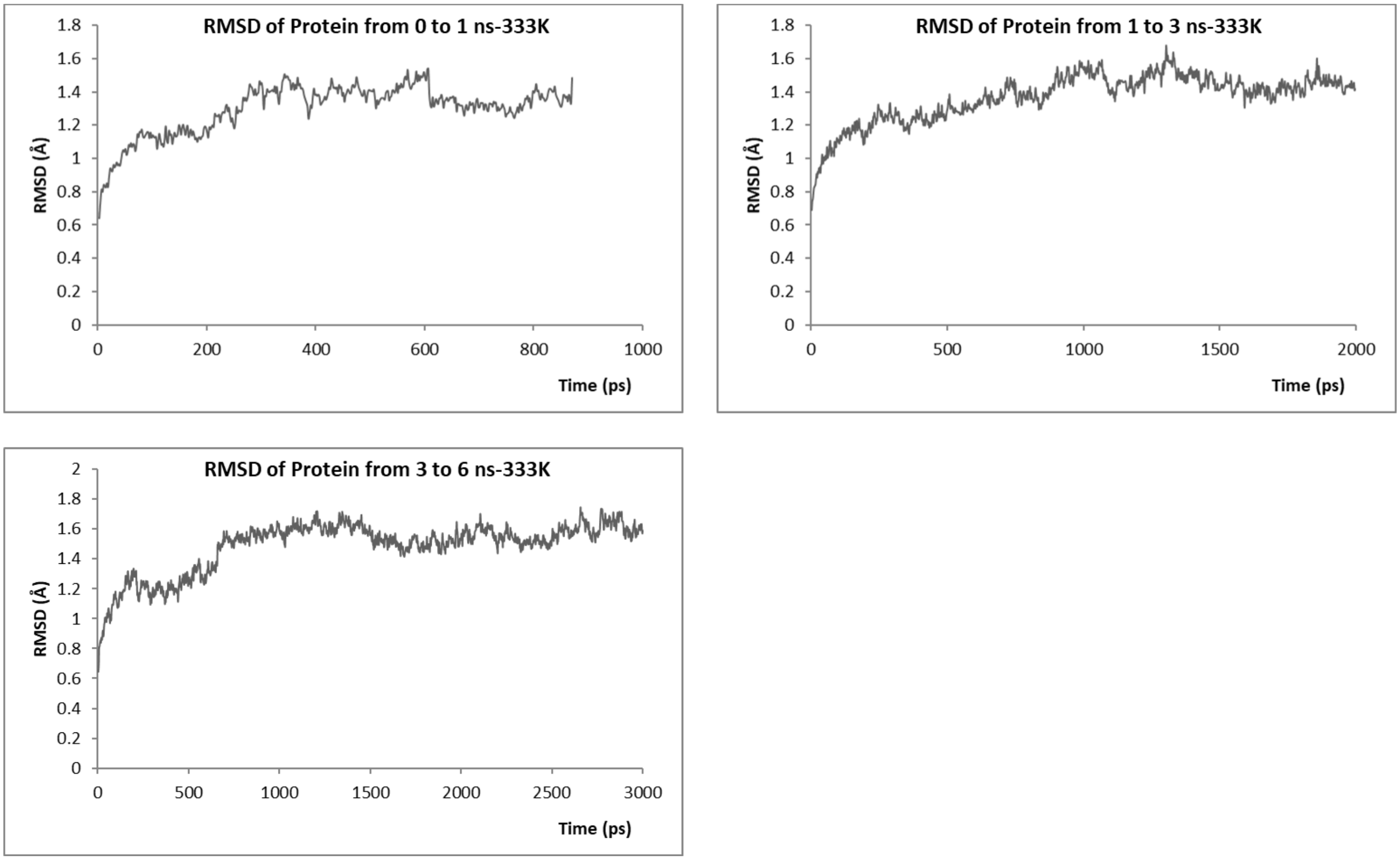

Figure S6: Root mean square deviation of protein from 0 to 1, 1 to 3, and 3 to 6 ns MD simulations of P450-TT enzyme as observed at 333 K.

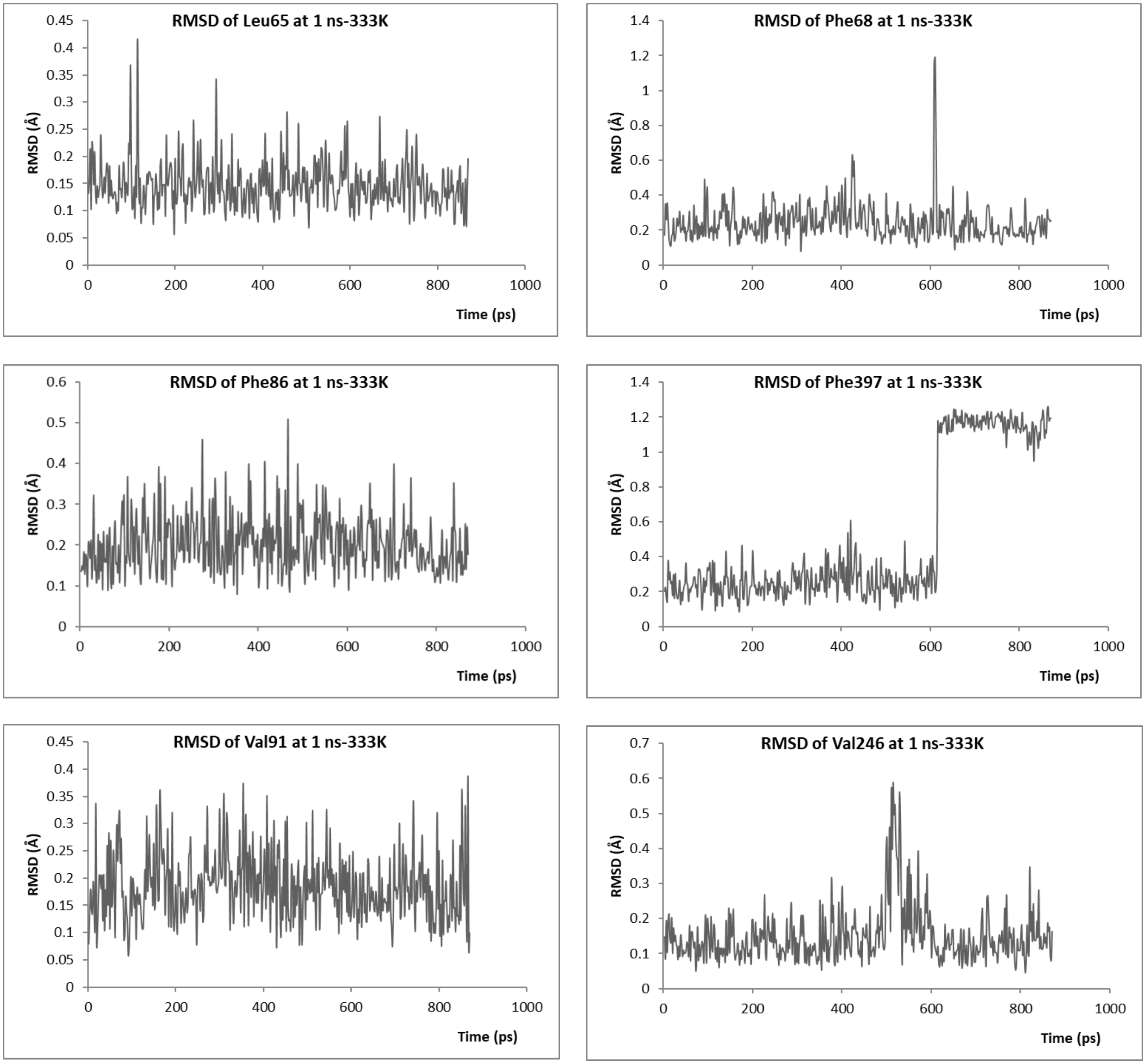

Figure S7: Root mean square deviation of Leu65, Phe68, Phe86, Phe397, Val91 and Val246 at 1 ns MD simulations of P450-TT enzyme as observed at 333 K.

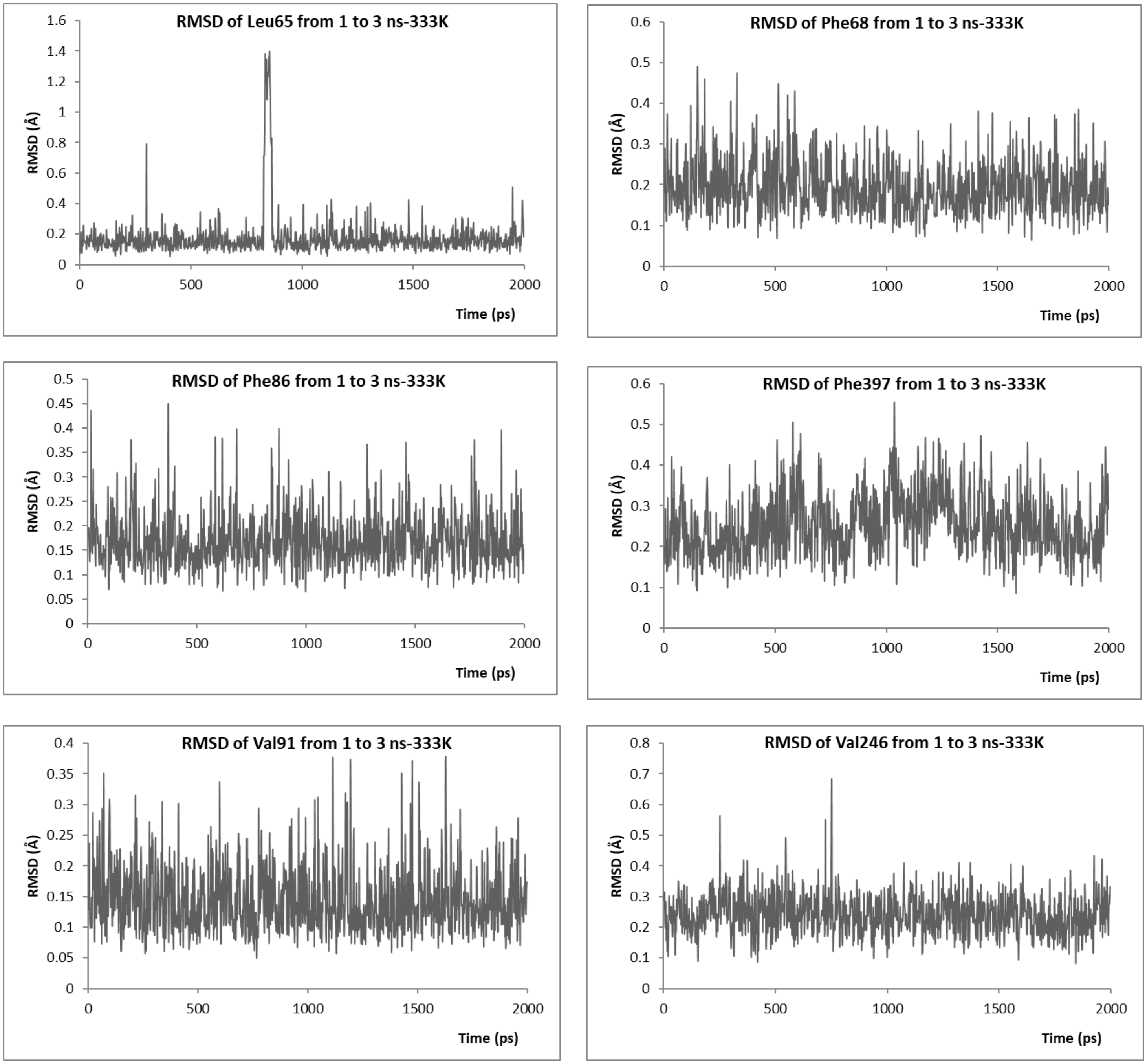

Figure S8: Root mean square deviation of Leu65, Phe68, Phe86, Phe397, Val91 and Val246 from 1 to 3 ns MD simulations of P450-TT enzyme as observed at 333 K.

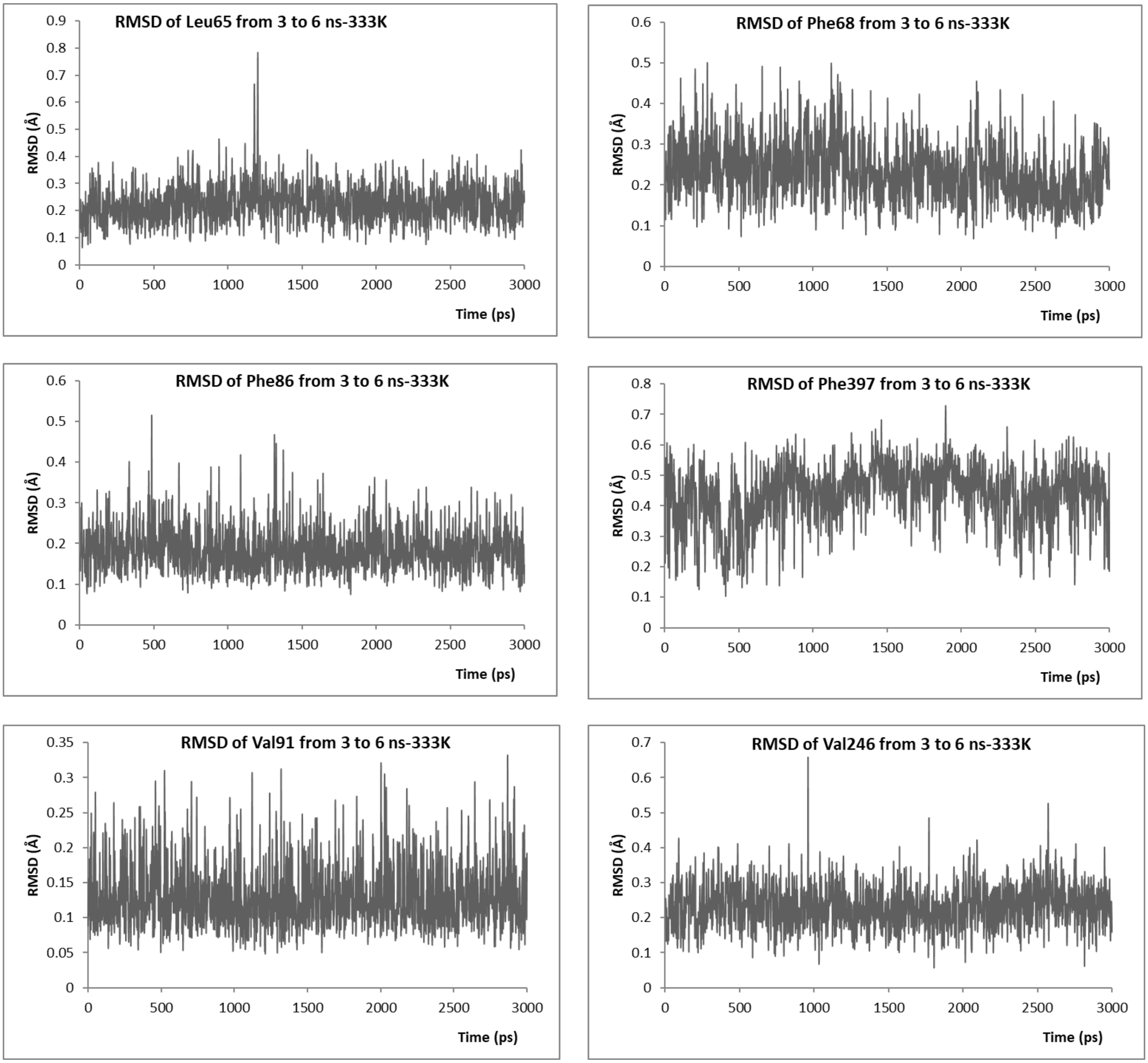

Figure S9: Root mean square deviation of Leu65, Phe68, Phe86, Phe397, Val91 and Val246 from 3 to 6 ns MD simulations of P450-TT enzyme as observed at 333 K.

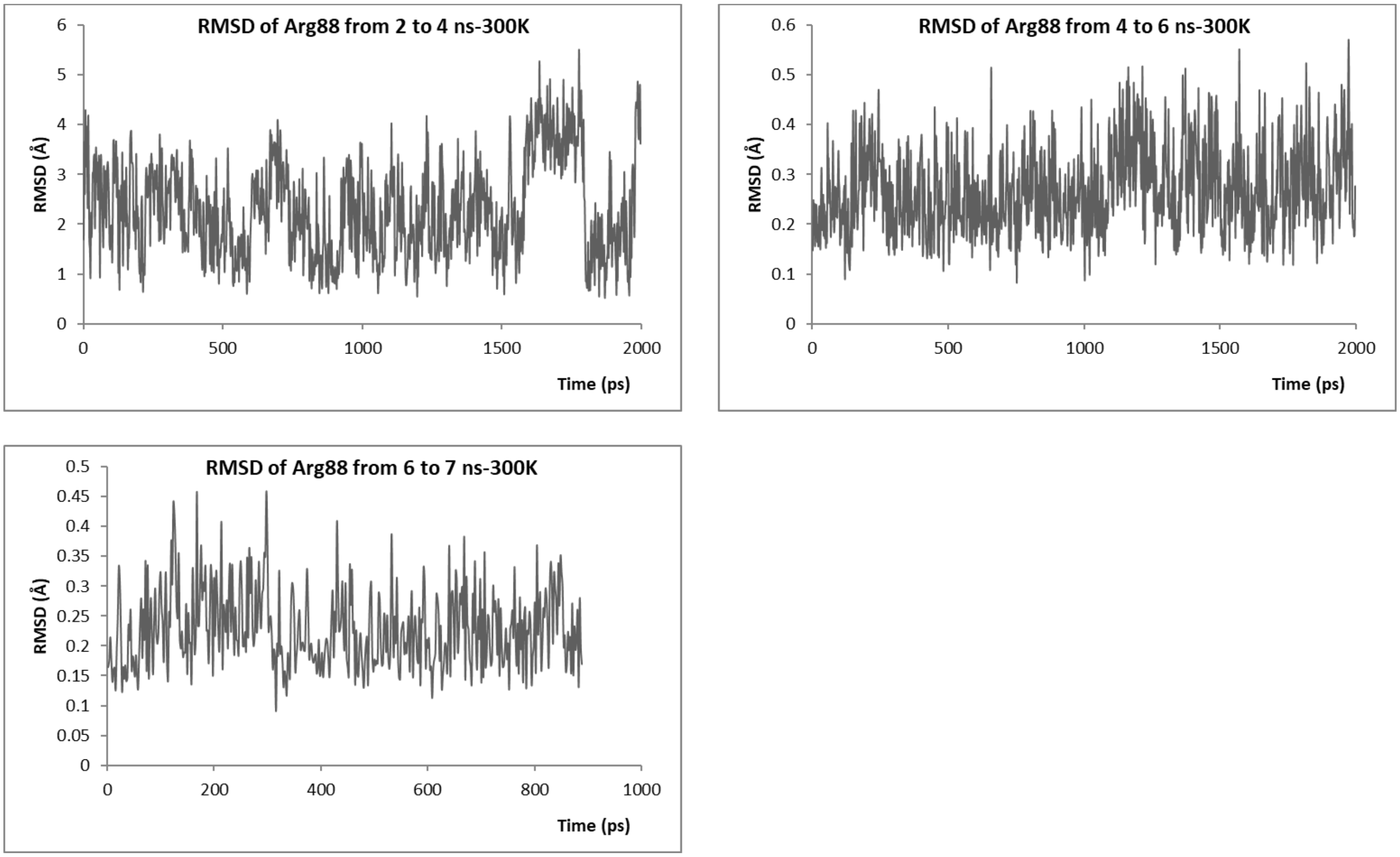

Figure S10: Root mean square deviation of Arg88 from 2 to 4, 4 to 6, and 6 to 7 ns MD simulations of P450-TT enzyme as observed at 300 K.

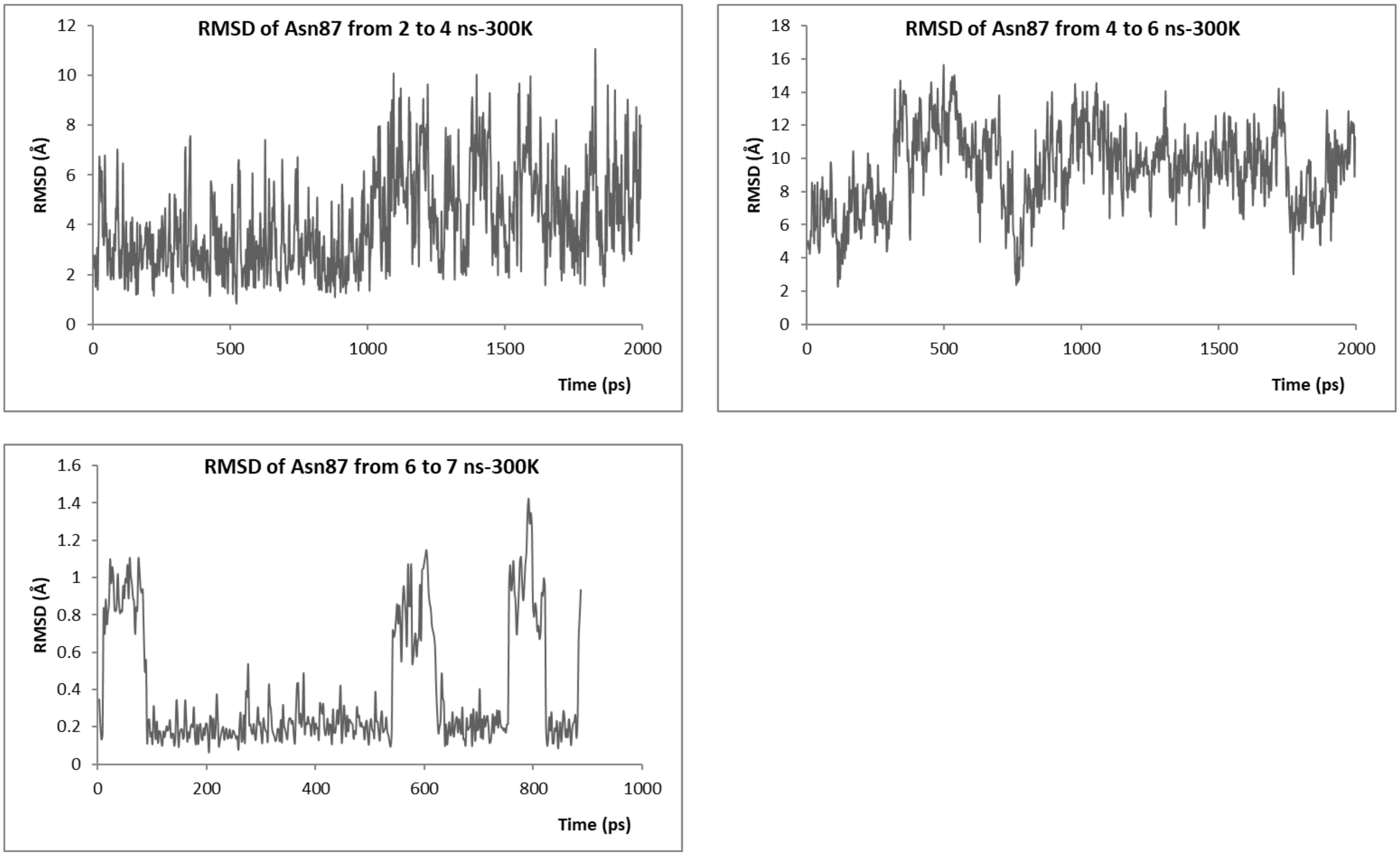

Figure S11: Root mean square deviation of Asn87 from 2 to 4, 4 to 6, 6 to 7 ns MD simulations of P450-TT enzyme as observed at 300 K.

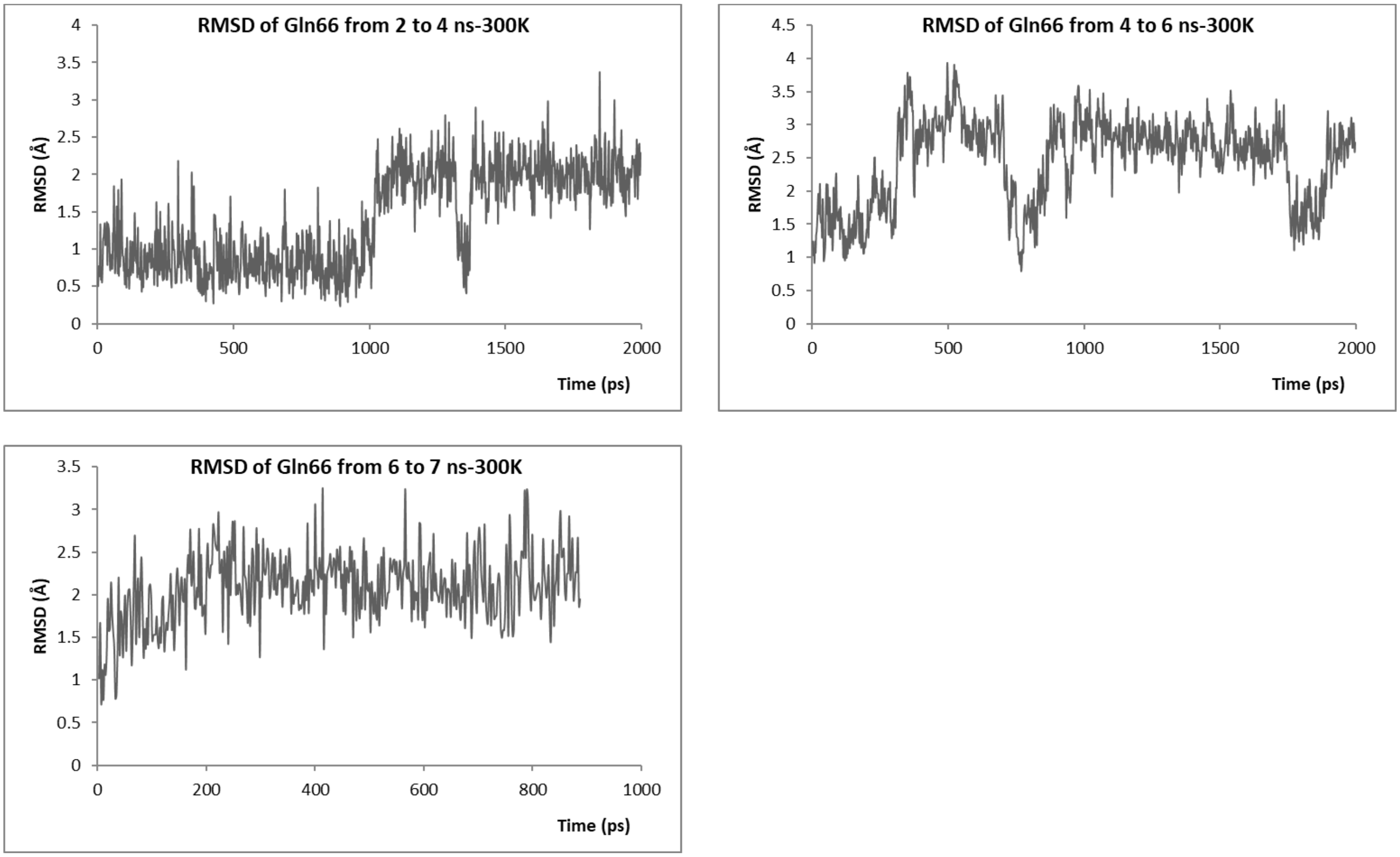

Figure S12: Root mean square deviation of Gln66 from 2 to 4, 4 to 6, and 6 to 7 ns MD simulations of P450-TT enzyme as observed at 300 K.

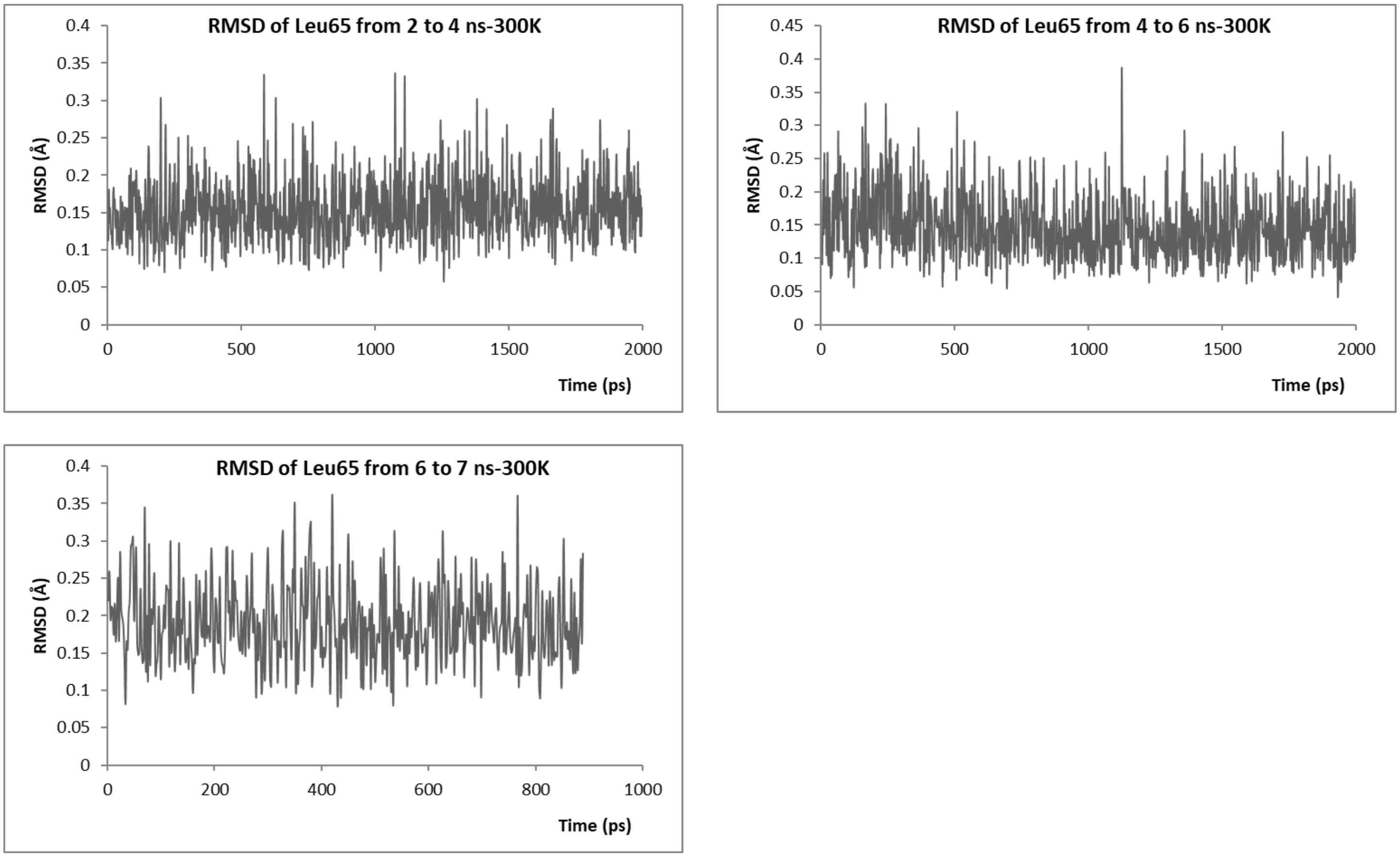

Figure S13: Root mean square deviation of Leu65 from 2 to 4, 4 to 6, and 6 to 7 ns MD simulations of P450-TT enzyme as observed at 300 K.

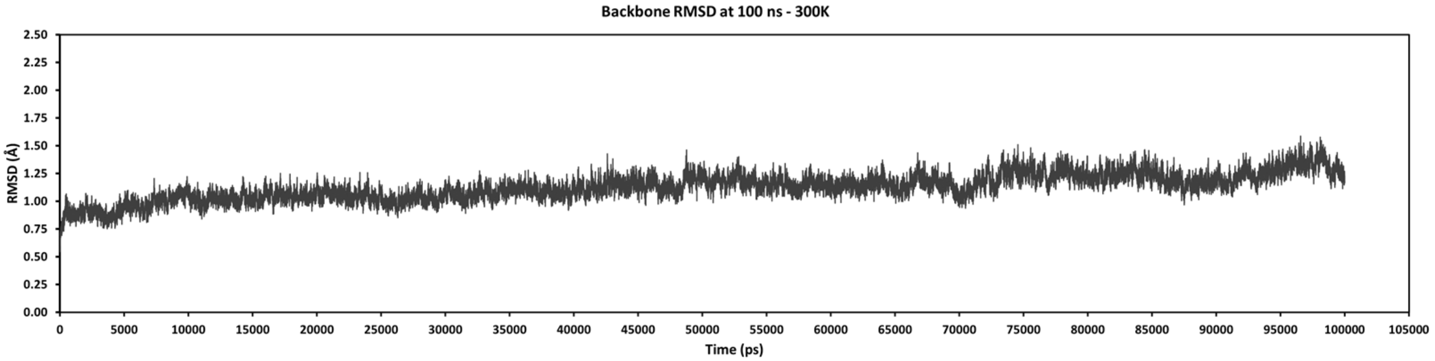

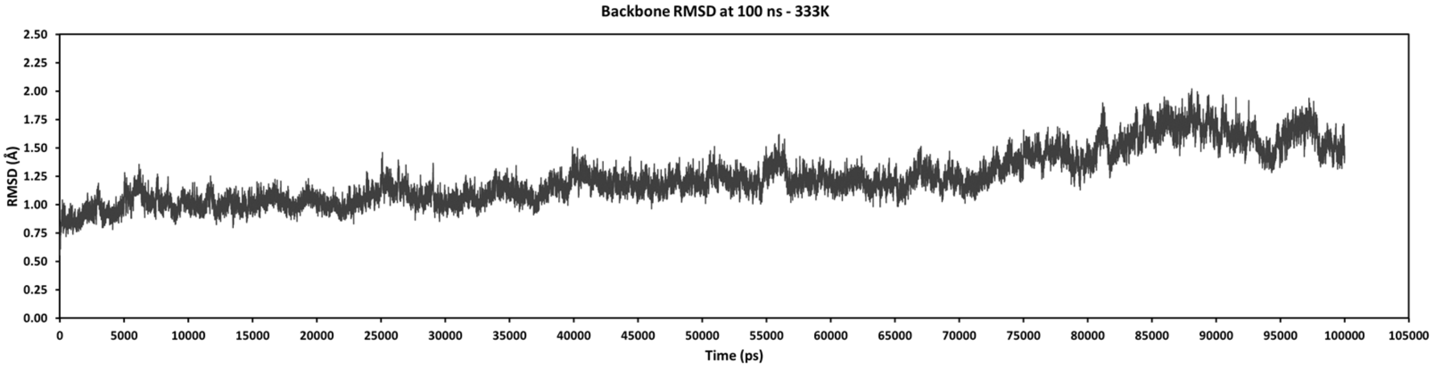

Figure S14: Backbone RMSD at 100 ns MD simulations of P450-TT enzyme as observed at 300 K and 333 K.

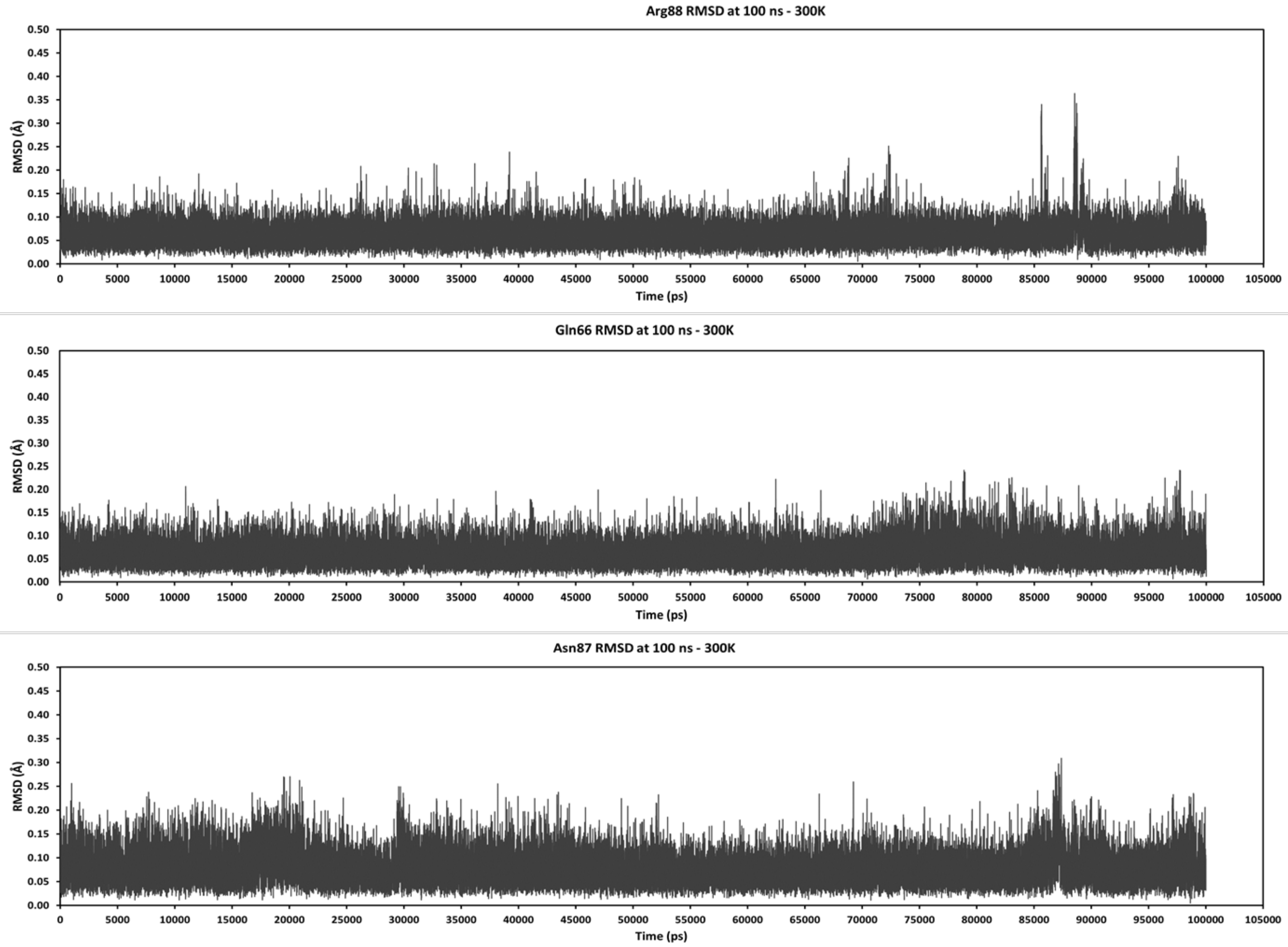

Figure S15: Root mean square deviation of Arg88, Gln66 and Asn87 at 100 ns MD simulations of P450-TT enzyme as observed at 300 K.

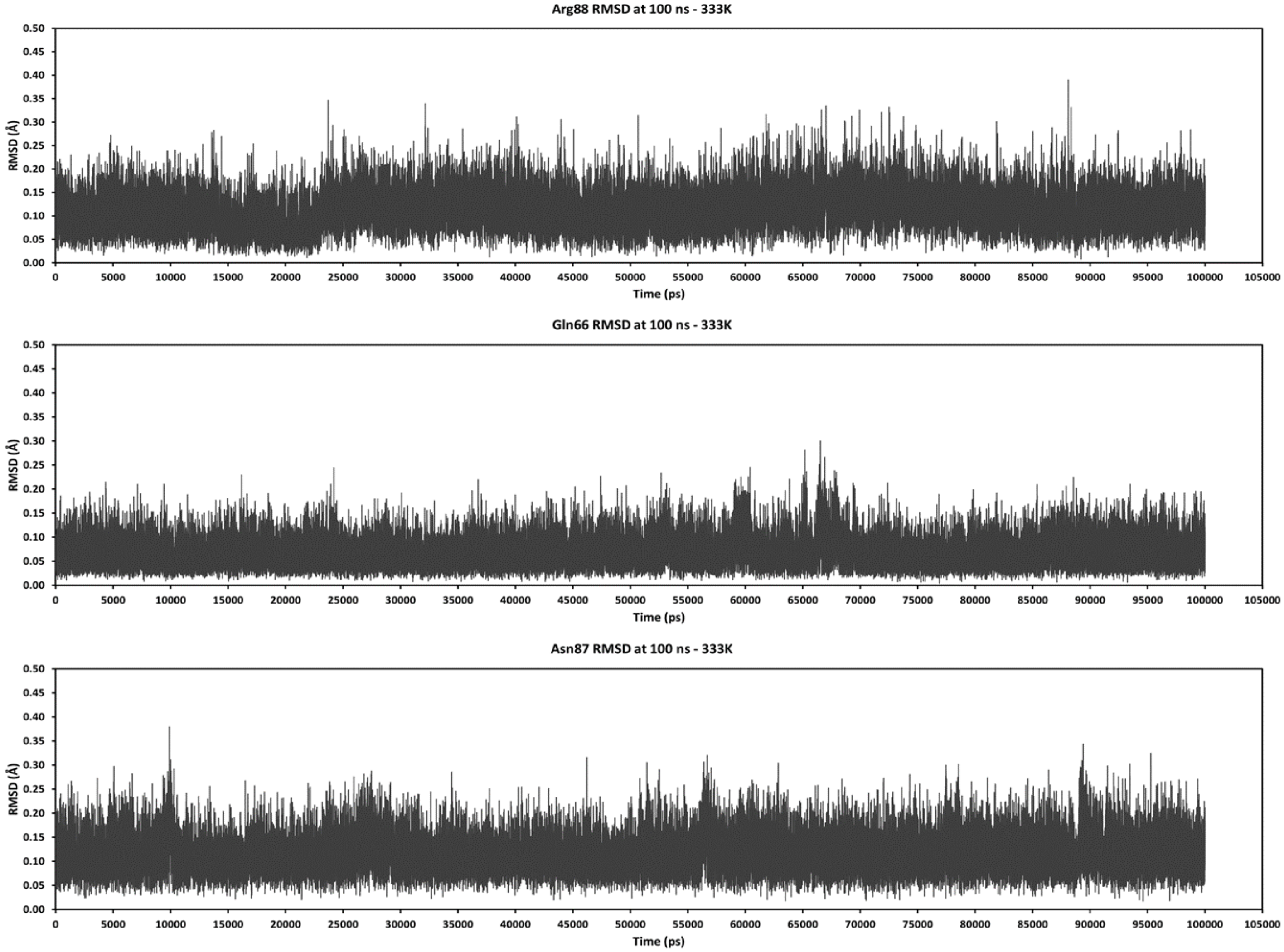

Figure S16: Root mean square deviation of Arg88, Gln66 and Asn87 at 100 ns MD simulations of P450-TT enzyme as observed at 333 K.

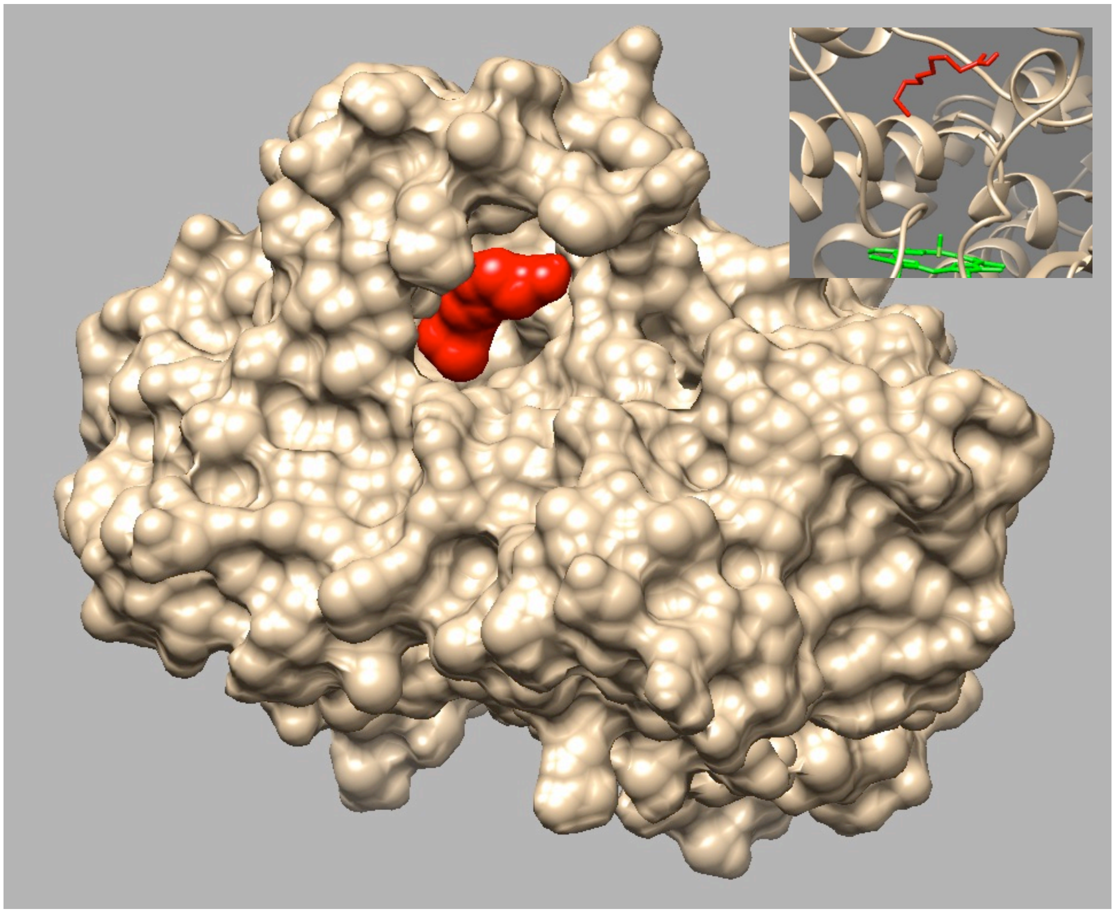

Figure S17. Illustration of the putative substrate decanoic acid docked within the substrate-binding channel of P450-TT conformation obtained at 100 ns of the 300 K MD Simulation. Inset: docking orientation 1 with binding affinity -3.5 kcal mol^{-1}.

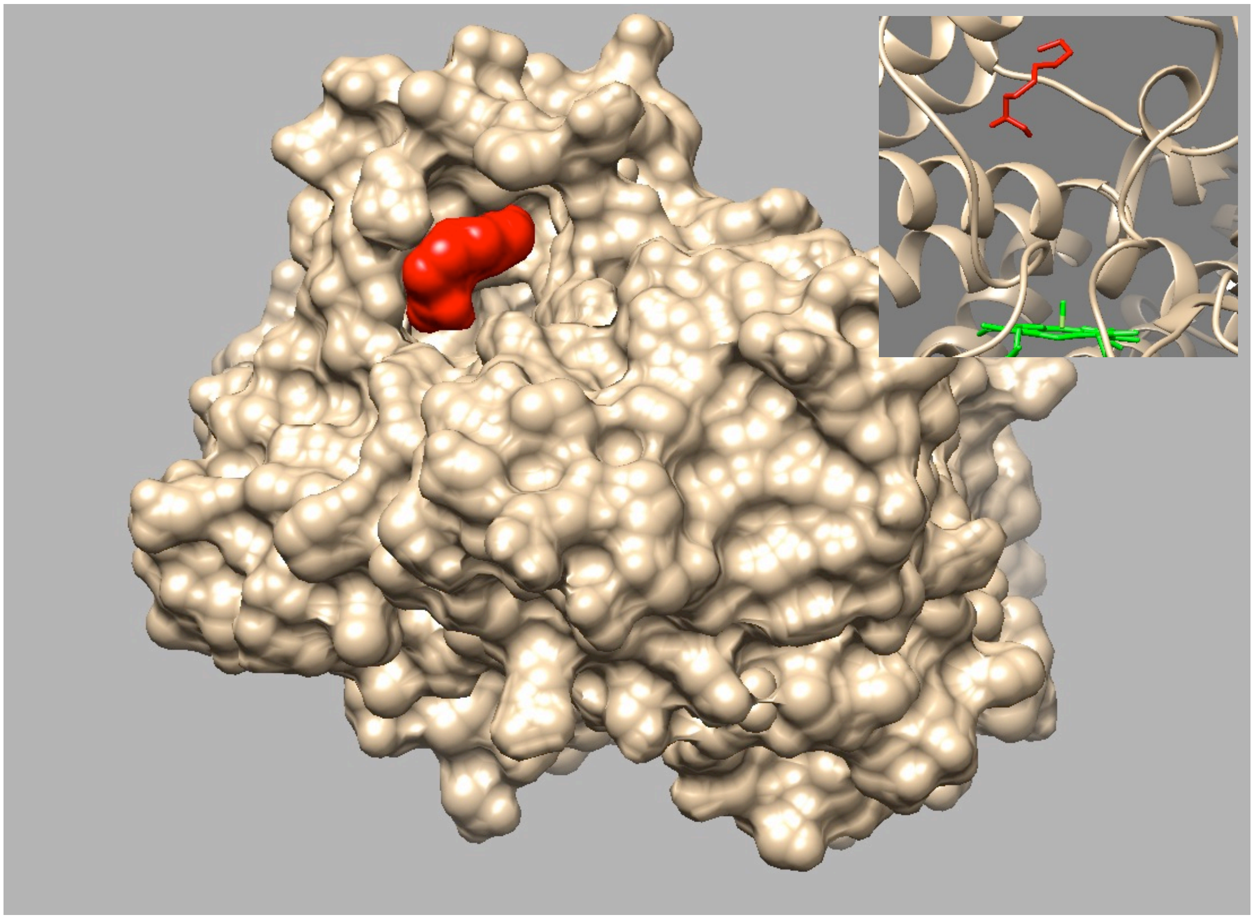

Figure S18. Illustration of the putative substrate decanoic acid docked within the substrate-binding channel of P450-TT conformation obtained at 100 ns of the 300 K MD Simulation. Inset: docking orientation 2 with binding affinity -3.3kcal mol^{-1}.
